# Electrochemical variability of natural mineral mixtures as electrode materials in Li-ion batteries

**DOI:** 10.1039/d6eb00037a

**Published:** 2026-07-21

**Authors:** John Donald Morley, Kathryn Hadler, Chandramohan George, Pablo R. Brito-Parada

**Affiliations:** a Department of Earth Science and Engineering, Imperial College London London SW7 2AZ UK john.morley18@imperial.ac.uk; b Dyson School of Design Engineering, Imperial College London SW7 2AZ London UK; c European Space Resources Innovation Centre (ESRIC), Luxembourg Institute of Science and Technology (LIST) Maison de l'Innovation 5 avenue des Hauts-Fourneuax L-4362 Esch-sur-Alzette Luxembourg

## Abstract

Natural mineral systems can be effectively used to make battery material supply chains more secure and sustainable. However, the impact of their multi-mineral electrochemistry must be considered for effective deployment. Mine tailings are shown as a potential sustainable natural mineral source. A simplified ternary system of pyrite, quartz and muscovite was examined for its trends in electrochemical performance. Mixing of minerals is shown to adversely affect charge transfer impedance (*R*_ct_) and diffusion coefficient (*D*_Li^+^_) values post-cycling; *R*_ct_ values are 10 times greater for mixtures than pure phase minerals, and *D*_Li^+^_ values are an order of magnitude lower than pure phase minerals. Within mixtures, the component of capacity from pyrite decays more rapidly than in a pure mineral phase. A comparison with two mine tailings reveals the inclusion of lesser amounts of pyrite in a majority silicate mixture to boost electrochemical performance, while the opposite leads to reduced performance. The results convey that a clear link between mineral composition and expected electrochemical performance is required to fully realise the benefits of mixed natural mineral sources for sustainable battery electrodes.

## Abstract 2

Trends in electrochemical performance for a ternary mixture of pyrite, quartz and muscovite are shown to be non-linear and complex.

## Graphical abstract

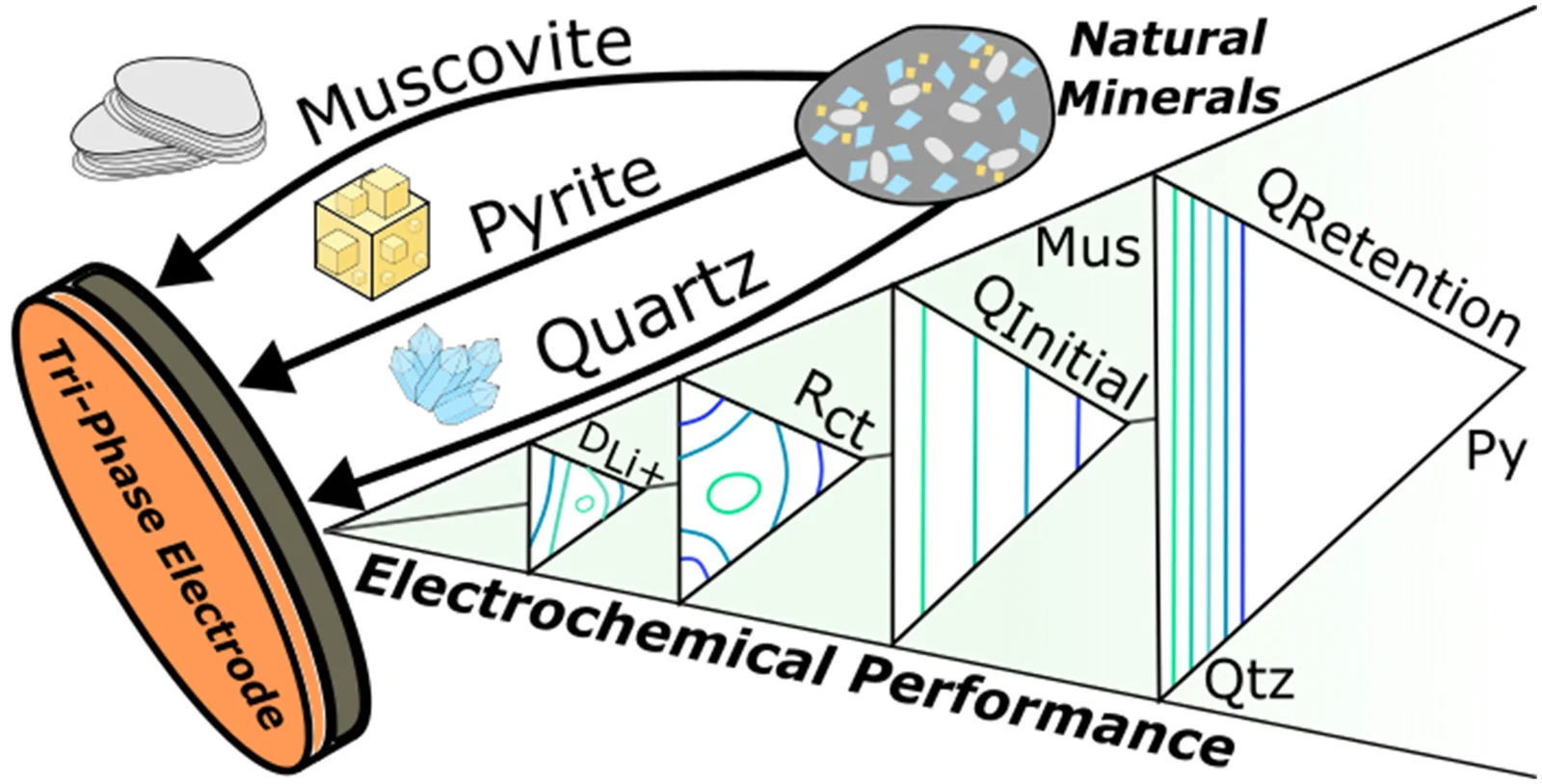

**Broader context**

Lithium-ion Batteries (LIBs) are essential for the forthcoming green energy transition. Secure supply chains are critical for the sourcing of active electrode materials required for the increasing demand for LIBs. However, strains on the production and supply chains of these materials are increasing, including monopolised supplies, high water and energy intensities and social and environmental issues during raw mineral extraction. The use of geospatially abundant natural minerals directly as active materials within battery electrodes could help alleviate these issues. The direct use of naturally sourced minerals is made challenging as they are a complex mixture of mineral phases with variable electrochemical responses. Within this work, we outline the electrochemical trends of a tri-phase mixture of the natural minerals: pyrite, quartz and muscovite for LIBs. Trends in capacity, impedance and lithium-ion diffusion are shown to be non-linear and variable. Mine tailings are used as an example of a mixed natural mineral system to provide real-world context. Understanding these electrochemical trends is a crucial step in the valorisation of natural minerals directly for LIBs, furthering the potential for future reduction in production and supply chain stressors of current battery materials.

## Introduction

Demand for batteries is expected to increase by 33% annually due to the green energy transition.^1^ The sourcing of current commercial battery materials, such as graphite, Lithium Iron Phosphate (LFP) and Nickel Manganese Cobalt Oxide (NMC), is complex for a multitude of reasons: high water intensities, illegal and child labour, monopolised supplies and high energy intensities due to the milling and heating of electrode materials.^2^ Despite these concerns, these materials are essential for the battery industry, for example, graphite is expected to account for 53.8% by mass of all mineral demand for energy storage by 2050.^3^ Governmental policies put further strains on already tight supply chains, such as China's export restrictions on rare earth elements, tungsten, and molybdenum in 2014.^4^ Increases in raw material costs for raw battery materials such as Li_2_CO_3_ by 830%, CoSO_4_ by 100% and NiSO_4_ by 60% in 2022 caused prices of prismatic cells for electric vehicles to increase by 30% and 50% for NMC and LFP, respectively, within the same year.^5^ One approach to address these issues is to use natural mineral sources for batteries due to their geospatial natural abundance and cheaper production costs due to minimised processing. Many natural minerals and mineral groups have been studied for use within batteries: stibnite,^6–8^ pyrite,^9–11^ clays^12^ such as halloysite^13^ and montmorillonite,^14,15^ phosphates^16^ and organic materials such as rice husks.^17,18^ However, intense chemical synthesis or processing to generate the desired electrochemical performance increases demand for energy, water and chemical supplies, creating further environmental issues. Sustainable sourcing of battery materials is becoming increasingly important;^19,20^ previous literature predominantly focuses on the sustainable production of already matured battery materials and systems such as graphite, hard carbon and lithium-sulphur batteries.^21^ However, a new focus is now placed on the utilisation of impure materials to benefit from their cheaper and more sustainable production, although there is typically a reduction in electrochemical performance.^22^ For these types of materials to be considered, a comprehensive assessment of sustainability and performance must be conducted.

Previous research has focused on mixing as a way to benefit from the preferential properties of different phases to combine their benefits. Mixtures of battery active materials, including Lithium Manganese Oxide (LMO), Lithium Cobalt Oxide (LCO), NMC, Nickel Cobalt Aluminium oxide (NCA) and LFP, generated from the recycling of Li-ion batteries, show the introduction of LFP to negatively impact electrochemical performance due to LFP's lower nominal voltage of ∼3.2 V.^23^ Mixing of conventional battery materials such as NMC and LCO has been shown to increase battery safety and rate performance,^24^ with higher upper potential limits of 4.5 V being reached.^25^ The use of mixtures has also been utilised to buffer the impact of volume expansion on electrochemical performance for SnO_2_,^26^ Sn^27^ and pyrite.^28^ The generation of unique particle structures by mixing has also led to increased electrochemical performances; the mixing of TiO_2_ and GeO_2_ in a 5 : 1 ratio created an opal honeycomb structure with an initial specific capacity of 1665 mAh g^−1^,^29^ while the mixing of NiO with Co_3_O_4_ created nanoflower structures with specific capacities of 1428.8 mAh g^−1^ after 100 cycles at 0.1 A g^−1^.^30^ Mixing has also been shown to enhance particle crystallinity, for example, mixing cobalt and tin oxalates,^31^ and to improve redox activity at higher potentials with the mixing of V_2_O_3_ and Li_3_PO_4_^32^ and the mixing of iron(ii) and iron(iii) phosphates.^33^ Direct use of natural minerals leads to the introduction of multiple minerals either as major or minor phases within the active material of a battery electrode. The blending of traditional cathode materials has been studied with spinel structures, olivine structures and NMC to combine the advantages of each material: high power and energy density with stable performance and lower costs.^34^ However, for minerals sourced naturally, there is no choice of mineral as the source will contain several major and minor mineral phases based on the local geology. Hence, the impact of mixing naturally sourced minerals commonly occurring together must be studied systematically to understand their interdependence on performance and to leverage their differing electrochemical benefits.

Mine tailings are an example of an abundant natural source of minerals and have previously been shown to have specific capacities >100 mAh g^−1^ after 100 cycles at 1C when directly used as anode material within Li-ion batteries.^35^ Storage of mine tailings generates a multitude of issues, from acid mine drainage^36^ to tailing dam failures.^37^ Upcycling of mine tailings has been studied to produce rare earth elements and for use as geopolymers,^38^ material for supercapacitors^39^ and batteries.^35^ Here, a comprehensive study of the impact of mixing minerals in a ternary system is provided, sampling the entire composition space, unlike previous research on ternary systems for batteries.^40^ A review of global mine tailings compositions is used as a basis to determine the minerals for the tri-phase system. These mixtures are then used to study the impact of mixing natural minerals as active materials on electrochemical performance. A simplified ternary mixture of pyrite, quartz and muscovite shows that electrochemical performance deteriorates more rapidly for charge transfer resistance (*R*_ct_) values, lithium-ion diffusion coefficient (*D*_Li^+^_) values and the pyrite contribution to capacity from differential capacity analysis compared to single mineral phases. Comparisons made with real mine tailings used as active battery electrode material highlight the variable impact of mineral type on battery performance as either a minor or major phase within the electrode.

## Methodology

### Mine tailings composition clustering analysis

A literature review of fifty-five scientific articles and reports was carried out, yielding 231 mine tailings compositions from 54 different mines and or mine tailings sites (SI workbook W1 for the compositions and original references and W2 for the mine/mine tailings site locations). The compositions were split into nine mineral categories: quartz, feldspar, micas (including chlorite), clays, carbonates, sulphates, pyrite, other sulphides, and metal oxides.

Hierarchical clustering was undertaken using the hierarchy module within Scipy.cluster library of Python3 to determine the number of clusters the data should fit.^41^ The agglomerative clustering module was used with the settings of three clusters, a Euclidean affinity and ward linkage to perform the clustering of the normalised and filtered mine tailings compositions.

### Material characterisation

Pyrite was obtained from Sigma Aldrich as a powder, while specimen crystals of quartz and muscovite were obtained from GEO Supplies Ltd. The quartz and muscovite crystals were ground to a fine powder and passed through a 53 μm sieve.

Particle size distribution was determined by laser diffraction using a Mastersizer 3000E from Malvern Panalytical. Micron-sized particle morphology and Electron Dispersion X-ray (EDX) spectroscopy were analysed using a TFS Quanta Environmental Scanning Electron Microscope (ESEM) at an accelerating voltage of 20 kV. Nano-sized particle morphology and crystallography were analysed using Transmission Electron Microscopy (TEM) with a JEOL STEM 2100Plus at an accelerating voltage of 200 kV. Elemental analysis was conducted using X-Ray Fluorescence (XRF) using an Epsilon 3 XRF from Malvern Panalytical Crystallographic analyses were undertaken using X-Ray Diffraction (XRD) with a Rigaku Miniflex 600 using an accelerating voltage of 30 kV and a 2*θ* angle range of 10° to 90°.

### Cell preparation

The working electrodes were produced by mixing 70 wt% of the active material at the given ternary mixture ratio, 20 wt% super P carbon black from Sigma Aldrich and 10 wt% poly(vinylidene fluoride) binder from MTI Corps in *N*-methyl-2-pyrrolidone solvent from Sigma Aldrich to form an electrode slurry with a solid mass of 0.25 g. For the pure phase pyrite, quartz, and muscovite, the mineral powders were directly used as active anode materials. For the mixtures, 21 mixtures of pyrite, quartz and muscovite, as the active material for the anodes, were produced with mineral compositions varying from 0% to 100% in 20% intervals. After mixing with an agate pestle and mortar, the slurry was uniformly spread at a thickness of 100 μm on 9 μm thick copper foil and dried in ambient conditions for 15 hours, followed by heating at 30 °C for 5 hours. The electrodes were cut with a diameter of 14 mm and weighed to determine their mass loading.

Half cells were manufactured in a UNIlab pro glovebox manufactured by MBraun in an Argon atmosphere with <0.1 ppm O_2_ and H_2_O. 2032 half cells were made using a lithium disc (thickness 600 μm), counter electrode, polyethene (PE) separator (thickness 8 μm), two 0.5 mm stainless steel spacers, a wavy spring and 100 μl of 1.0 M LiPF_6_ (dissolved in EC and DEC of equal volumes) as the electrolyte. Cells were crimped at a pressure of 0.8 tm^−2^, with electrochemical testing undertaken after a 1-hour wait.

### Electrochemical characterisation

Electrochemical testing was performed at room temperature on a PARSTAT MC Multi-Channel and a Landt battery cycler. Galvanostatic cycling of the batteries was performed at different C-rates. The C-rate for each pure mineral phase was determined based on its capacity after cycling at 0.05 A g^−1^. Cyclic Voltammograms (CVs) were taken at a scan rate of 0.1 mV s^−1^ within a 0–3 V potential range. Electrochemical Impedance Spectroscopy (EIS) measurements were conducted from 0.1 Hz to 100 kHz pre-cycling and 0.1 mHz to 100 kHz post-cycling, on cells as assembled and fully charged cells, with a 1-hour waiting time between galvanostatic charging and taking the EIS measurement. Equivalent circuit fitting of EIS data was conducted using the ZView 4 software. Testing of each ternary mixture and pure phase sample included an initial EIS spectrum followed by cycling at 1C for 100 cycles, followed by a final EIS spectrum. The C-rate was determined for each mixture by a mass-weighted linear average of the specific capacities of the pure phase minerals within the mixture. Experimental results for the ternary mixtures were repeated if the values determined were outliers or did not follow the trend of the other mixtures within the ternary system to provide validity for the trends observed. Galvanostatic Intermittent Titration Technique (GITT) was undertaken on all three pure-phase mineral samples by following a 7-minute current pulse at 1 C with a measure of the potential for 10 hours within an open circuit.

## Results

### Mine tailings composition analysis

The weight percentage distribution for the 9 main mineral groups studied for mine tailings composition is shown in Fig. 1A. Quartz was the most abundant mineral group by wt% with an average wt% of 43.8%. All other mineral groups had wt% <20%, with sulphides accounting for only 1.1% of the mine tailings composition. From Fig. 1B, the geospatial spread of the published mine tailings compositions can be seen. Mine tailings compositions are reported from all 6 inhabited continents, with a bias towards compositions from Canada, Europe, and China. This is of interest as Europe does not currently undertake extensive mining activities, unlike other regions such as South America. This contrast between mine tailings studied and mining activity is likely due to a collection and research bias.

**Fig. 1:**
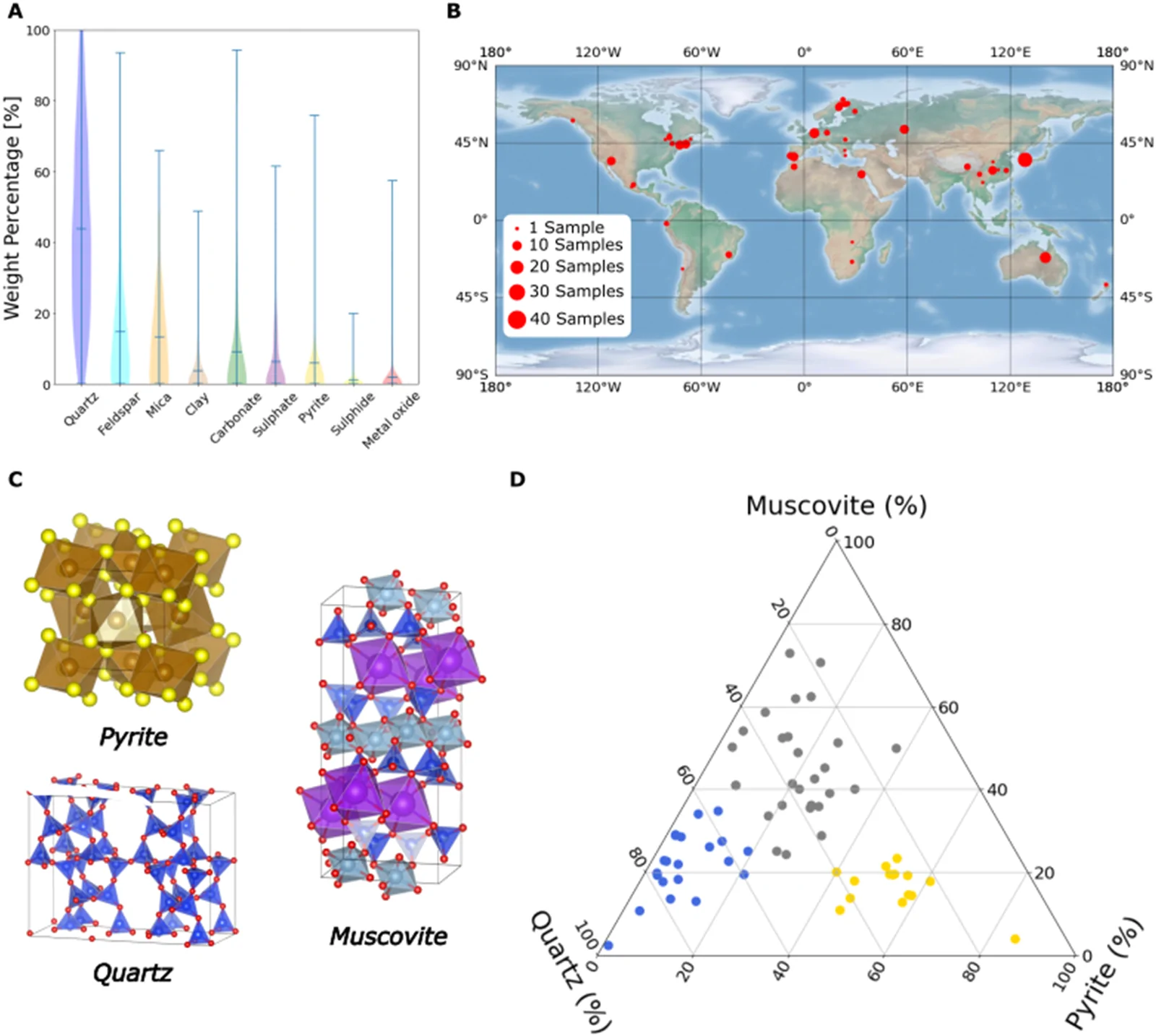
A literature review of 231 mine tailings compositions. (A) Violin plot of 9 mineral groups and their weight percentages within the total 231 mine tailings compositions. (B) Map highlighting the mine locations of the mine tailings compositions. (C) Crystallographic structures of pyrite, quartz, and muscovite were produced using the VESRA software.^43^ (D) Ternary diagram, highlighting the clustering of pyritic-rich mine tailing compositions.

A simplified mineralogical system is required to enable a more systematic approach to identify the roles and impacts of minerals within mixtures for battery electrode performance, removing the complexities of mine tailings, including variability in composition, morphology and surface alterations (weathering) and its impact on electrochemical performance.^35^ Clustering analyses were undertaken to simplify the complex mineralogical compositions of mine tailings, shown in Fig. 1A. The dendrogram of the hierarchical clustering is shown in Fig. S1. The red line shows the region with the greatest vertical line length, as it cuts three branches of the dendrogram; it is inferred that the data should be clustered into three groups. From the mineral abundances and clustering analysis, the three minerals quartz, muscovite and pyrite were chosen as the pure minerals for the ternary system. Quartz was chosen as it is the most abundant mineral, while muscovite was chosen as it is the more generic form of mica. Feldspars were not chosen, despite having an average wt% larger than the micas, as they have a similar tectosilicate structure to quartz, while muscovite has a different layered phyllosilicate structure, as shown in Fig. 1C. As battery performance and electrochemistry are a function of active material crystallography^42^ it is of greater interest to study two silicates of differing crystal structures. Pyrite was chosen as the third mineral as its electrochemistry has been thoroughly studied with laboratory-synthesised samples; hence, its electrochemical mechanisms are thoroughly understood.

After filtering and normalisation of the mine waste compositions, the agglomerative clustering was undertaken on 64 mine tailings compositions and yielded the results shown in Fig. 1D. The three clusters are highlighted: yellow for pyrite-rich, blue for quartz-rich and pyrite-poor and grey for muscovite-rich and pyrite-poor. This grouping is a function of the geology of the ore deposits and the subsequent processing strategy employed, *e.g.*, what minerals were there before processing, and which were selectively removed or enhanced.

### Material characterisation

The particle size distribution of the three pure mineral phases, after grinding and sieving, is shown in Fig. 2A. The P_80_ (size by which 80% of particles by volume are smaller) values are 31.1 μm, 66.9 μm and 86.4 μm for pyrite, quartz and muscovite, respectively. Particle size and Particle Size Distribution (PSD) are known to strongly influence battery electrochemical performance.^44^ However, the impact is likely to be minimal as the effect has only been shown to be significant for nano-sized particles for pyrite^45^ and silica^46^ and not micron-sized particles, as the pure mineral phases are shown to be. Fig. 2B, C and D show SEM images of the pre-cycled electrodes of pyrite, quartz, and muscovite, respectively. Elemental maps from EDX are provided in Fig. S2, S3 and S4, respectively, for pyrite, quartz, and muscovite. The SEM and corresponding EDX map of the 60 wt% pyrite and 40 wt% quartz electrode in Fig. S5 and S6 show a homogeneous spread of both quartz and pyrite as Si, representative of quartz and Fe, representative of pyrite, are well spread within the SEM image. The pyrite and quartz particles are both jagged in morphology. The muscovite particles are shown as flat flakes, forming this morphology due to their perfect basal cleavage. All the flakes align so that the basal plane of the particles lies flat on the copper foil of the electrode. These differences in particle morphology must be considered as they have a direct impact on electrolyte infiltration, ion diffusion and electrochemical kinetics.^47^ The quartz and muscovite were powdered from natural crystals, hence XRF was undertaken to determine any elemental impurities. The XRF results for quartz shown in Fig. 2E highlight Fe and Cr as minor impurities, while muscovite has a greater number of elemental impurities (Fe, P, Cl, Na, Mg, S and Cr). The greater number of elemental impurities within muscovite, shown in Fig. 2F, is noted as it can more readily substitute metal cations within its metal octahedra, where potassium is typically stored.^48^

**Fig. 2:**
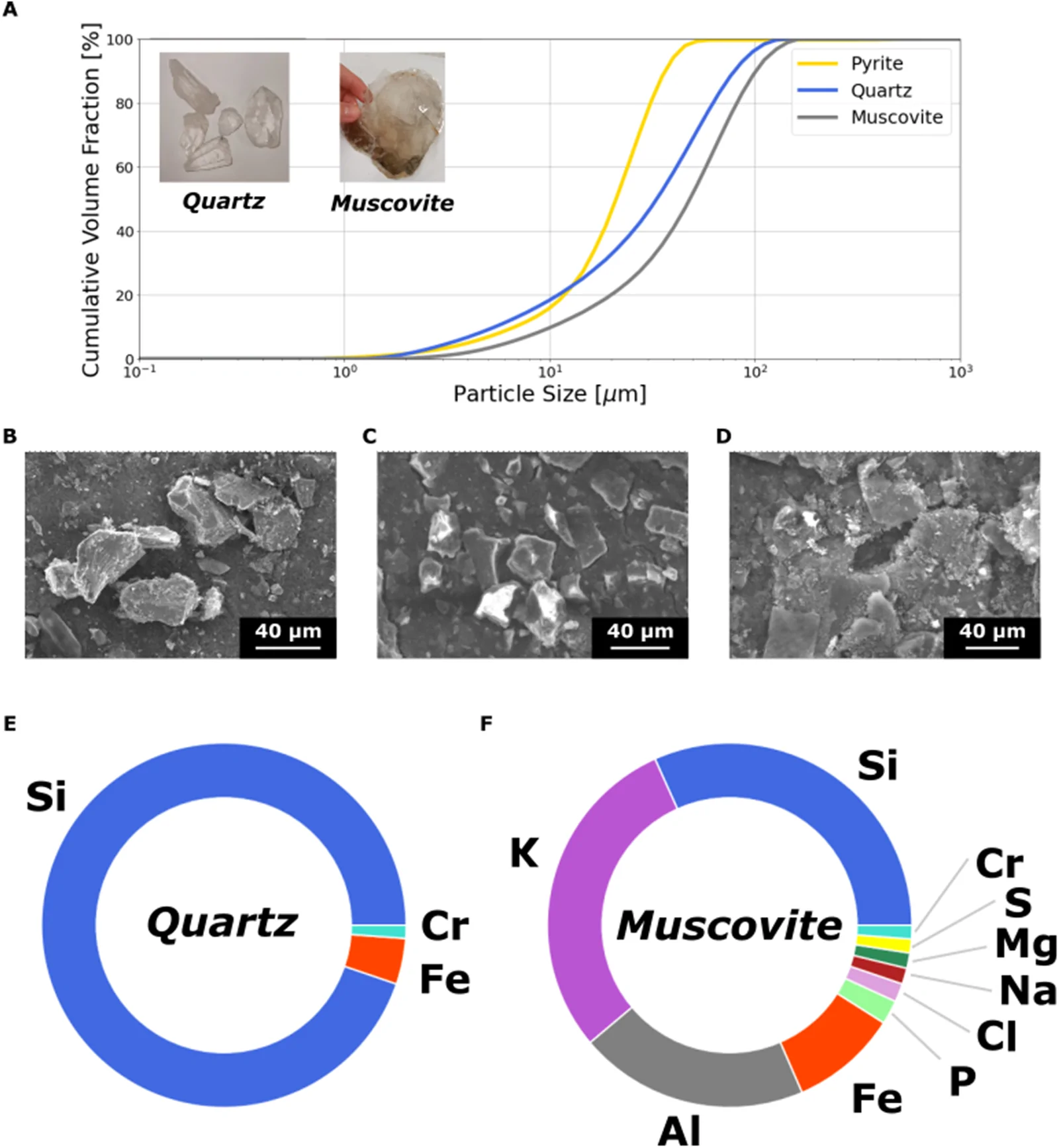
Material characterisation of three pure mineral phases. (A) Particle size distribution of pyrite, quartz and muscovite. Insert to show the original crystals of quartz and muscovite. (B) SEM image of the pyrite electrode. (C) SEM image of the quartz electrode. (D) SEM image of the muscovite electrode. (E) XRF elemental analysis of quartz (elements > 1 wt% shown). (F) XRF elemental analysis of muscovite (elements > 1 wt% shown).

### Electrochemical performance

#### Pure phases

Specific capacities from galvanostatic cycling tests are shown at a charge rate of 1C in Fig. 3A and 0.1C in Fig. S9. At 1C, pyrite shows the greatest initial specific discharge capacity of 860 mAh g^−1^ near the theoretical maximum of 894 mAh g^−1^ and comparable to values within the literature.^45^ The capacity rapidly declines to a minimum value of 149 mAh g^−1^ at the 64^th^ cycle. Rapid decreases in the specific capacity of pyrite are common and attributed within the literature to large volume expansions,^49^ formation of polysulphides and the polysulphide shuttling effect^50^ and non-circular reaction mechanism pathways^51^ during cycling. In contrast, quartz and muscovite, after their first cycle (initial specific discharge capacities of 168 mAh g^−1^ and 179 mAh g^−1^, respectively), retain a consistent specific discharge capacity of 80–100 mAh g^−1^. The similarity in their specific capacity values during cycling despite their difference in particle size, distribution and morphology, shows the silicate mineralogy to be the main control on specific capacity values measured. These values are significantly lower than the theoretical specific capacity of silica, 1965 mAh g^−1^,^52^ highlighting a severe underutilisation of the potential capacity of these natural silica-structured minerals. The sudden decrease in capacity during the first cycle for silicates is attributed to the formation of a Solid Electrode Interface (SEI) and the irreversible reaction of silicate to silicon and lithium oxide during the first lithiation of silicate minerals. At a 0.1C current rate, the initial discharge-specific capacities are very similar to those of cycling at 1C, as shown in Fig. S9. The performance of the quartz and muscovite is similar after 100 cycles when cycled at 0.1C or 1C. However, the capacity retention for the pyrite is improved when cycled at 0.1C as the specific discharge capacity after 100 cycles is 235 mAh g^−1^, a 45% increase in capacity compared to cycling at 1C. All three minerals show exceptional rate performance up to charge rates of 10C as shown in Fig. 3B. Both quartz and muscovite are shown to have specific discharge capacities of ∼100 mAh g^−1^ at 0.1C after cycling at 10C for 5 cycles, with specific discharge capacities of 40–50 mAh g^−1^. After returning to 0.1C after cycling at the higher charge rates of 0.5/1.0/5.0/10.0C the pyrite had a 45.9% decrease in specific discharge capacity. However, this is in line with the capacity decay seen when cycled at both 1C and 0.1C hence the rate performance is deemed good.

**Fig. 3:**
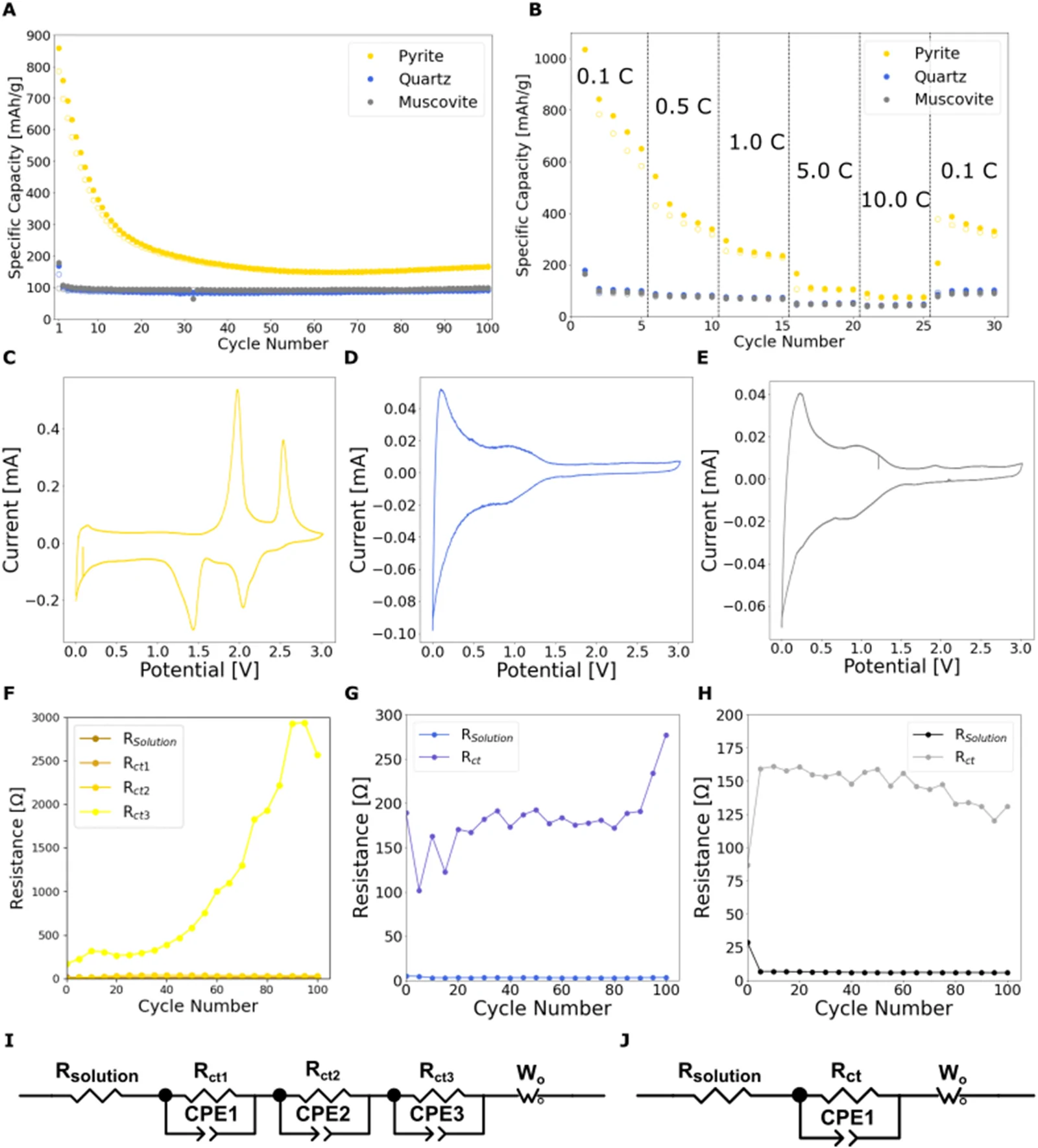
Electrochemical performance of pure phase pyrite, quartz, and muscovite within Li-half cells. (A) Galvanostatic cycling performance at 1C charge rate. (B) Rate test of the three pure phases. (C) CV at 0.1 mV s^−1^ of the pyrite phase. (D) CV at 0.1 mV s^−1^ of the quartz phase. (E) CV at 0.1 mV s^−1^ of the muscovite phase. (F) Equivalent fitted circuit impedance values for pyrite. (G) Equivalent fitted circuit impedance values for quartz. (H) Equivalent fitted circuit impedance values for muscovite. (I) Equivalent circuit used for modelling of pyrite EIS spectra. (J) Equivalent circuit used for modelling of quartz and muscovite EIS spectra.

CVs of the first cycle, at a scan rate of 0.1 mV s^−1^, are shown for all three mineral phases in Fig. 3C, D and E. The pyrite CV in Fig. 3C shows two anodic peaks at ∼1.95 V and ∼2.52 V and two cathodic peaks at ∼2.04 V and ∼1.43 V, attributed to the (de)lithiation of pyrite.^53^ An anodic peak <0.4 V is seen in both CVs for quartz and muscovite (∼0.37 V and ∼0.24 V, respectively), attributed to the lithiation of silicon formed from the silicate.^54^ An additional weaker anodic peak at ∼1 V for both quartz and muscovite is assigned to the reversible reaction of silicon to silicate.^55^ An additional minor cathodic peak is seen at ∼1.94 V for muscovite in Fig. 3E. The redox mechanism of this peak is difficult to decipher but is accounted for by the leaching of K^+^ or Al^3+^ ions from the muscovite crystal structure.^56^ The cathodic peak at ∼0.91 V for both quartz and muscovite is attributed to the reversibility of the silicon-silicate reaction.^55^ Fig. S8 shows the CV of a 60% pyrite, 40% quartz mixture electrode. Peaks are seen for both pyrite and quartz, showing both minerals to be electrochemically active within the mixed electrode. Galvanostatic (dis)charge curves, provided in Fig. S7, for the pyrite, quartz, muscovite, and 60% pyrite and 40% quartz mixture, show voltage plateaus at corresponding potentials to the peaks noted in the CVs. For the pyrite and 60% pyrite and 40% quartz mixture, the decay in capacity is ascribed to the decrease in the discharge plateau at ∼2.0 V and both plateaus during charge at ∼1.8 V and ∼2.5 V, occurring rapidly after the 10^th^ cycle. The more rapid decrease in the voltage plateau at ∼2.0 V than at ∼1.4 V during discharge shows the decay in discharge capacity to be dominated more by the decrease in the intercalation mechanism rather than the conversion mechanism of the pyrite. For both quartz and muscovite, after the initial first cycle, the (dis)charge curves show minimal change in shape, showing the electrochemical mechanism for the silicates to be consistent after their first cycle. The voltage plateau at ∼0.8 V seen in Fig. S7B and D for the quartz and 60% pyrite and 40% quartz mixture only during the first cycle is attributed to the reduction of silicate to silicon and formation of SEI.^57^ For the 10^th^, 50^th^ and 100^th^ cycle of the quartz and muscovite, the (dis)charge curves are sloped at potentials <1.0 V due to the lithiation of the silica structures and likely small amounts of silicon formed during the initial cycle.^35^

EIS were collected every 5 cycles for 100 cycles when cycled at 1C for each pure mineral phase (Fig. S10). The spectra were fit to their respective equivalent circuit models (Fig. 3I and J) with the impedances of their resistive components shown against cycle number in Fig. 3F–H. Different equivalent circuits were used for pyrite and the silicate minerals to account for the difference in the shape of their EIS spectra (Fig. S10). For the pyrite, the same equivalent circuit was used as that previously used for pyrite-rich mine tailings^35^ due to the complex arcing shape of the EIS spectra in the Nyquist space at low frequencies. For the silicate minerals, however, a modified Randles circuit was used^58^ as the EIS spectra exhibited the typical shape in the Nyquist space of a single semi-circle at high frequencies, followed by a linear Warburg region at low frequencies. The difference in impedance response is due to the differences in mineral crystallographic structure and subsequent difference in electrochemical behaviour during cycling between pyrite and the silicate minerals. The EIS spectra for pyrite were fit to four separate components with respective impedance values: *R*_solution_, *R*_ct1_ and *R*_ct2_, which all remained constant during cycling, and *R*_ct3_. The impedance assigned to the *R*_ct3_ is shown to increase exponentially from values of 268 Ω at cycle 20 to 2939 Ω at cycle 95. Both quartz and muscovite exhibit *R*_ct_ values <300 Ω. However, opposite trends are observed as quartz's *R*_ct_ values gradually increase from cycle 0 to 90 and then rapidly increase while muscovite's *R*_ct_ value gradually declines from 159 Ω at cycle 5 to 120 Ω at cycle 95. In addition, GITT was undertaken for the first charge and discharge of the pure phase pyrite, quartz, and muscovite electrodes to accurately determine initial *D*_Li^+^_ values for each of the pure phase minerals; the methodology and results are provided in SI S3.1. During the first discharge, the *D*_Li^+^_ values are shown to be approximately 10^−14^–10^−15^ cm^2^ s^−1^ for all three minerals and during initial charging to be within the same range, consistent with values for lab-synthesised pyrite^59^and natural silicate minerals.^35^ The *D*_Li^+^_ values are shown to be consistent despite the difference in PSD and particle morphology of the pure phase minerals, showing both factors to have minimal impact on electrochemical kinetics. The overpotential during the first discharge, seen in Fig. S11–S13, has low values of 0.1–0.3 V for quartz and muscovite but a greater value of 0.78 V for pyrite. This is accompanied by a lesser internal resistance with values <1000 Ω for pyrite but from 750 Ω to 2750 Ω for quartz and muscovite. During the first initial charge, the internal resistances are low for pyrite <700 Ω, while for quartz and muscovite, they rise after their initial lithiation plateau at potentials less than 1 V to values of 17 kΩ and 9 kΩ, respectively.

#### Ternary mixtures

For all 21 ternary mixtures, the calculated results and analyses from the galvanostatic cycling and EIS measurements are provided in SI workbook W3. The Key Performance Indicators (KPI) studied from the galvanostatic cycling were the initial specific discharge capacity and capacity retention after 100 cycles at 1C; Fig. 4 consolidates the data from workbook W3 into key trends across the ternary diagram space. For the initial specific discharge capacity, the contours of Fig. 4A show a linear trend with specific capacity increasing from <100 mAh g^−1^ at the 0 wt% pyrite region to >800 mAh g^−1^ at the pure pyrite region. Variable ratios of quartz and muscovite are not shown to impact initial specific discharge capacity, with only the ratio of silicate mineral (muscovite and quartz) to pyrite determining the trend. As with the pure mineral phases, specific capacity values are shown not to vary between the two silicate minerals despite their difference in particle size, distribution and morphology; hence, these factors are shown to have minimal effect on the specific capacity measured, with the differences in mineralogy predominantly controlling the trends measured. For capacity retention, a different trend is observed as there is a sharp decline in capacity retention from >90% to <30% from 0 wt% to 20 wt% of pyrite. Pyrite is shown to cause a dramatic drop in capacity retention even at low weight percentage values. Quartz and muscovite are shown not to have an impact on capacity retention as the contours are sub-parallel to the quartz-muscovite isoline. Initial coulombic Efficiency (CE) values, shown in Fig. S16, show a smooth increase in initial CE as pyrite content increases from 60% to 90%, highlighting the irreversibility of the initial (de)lithiation of silicate minerals as they lithiate to form silicon.^60^

**Fig. 4:**
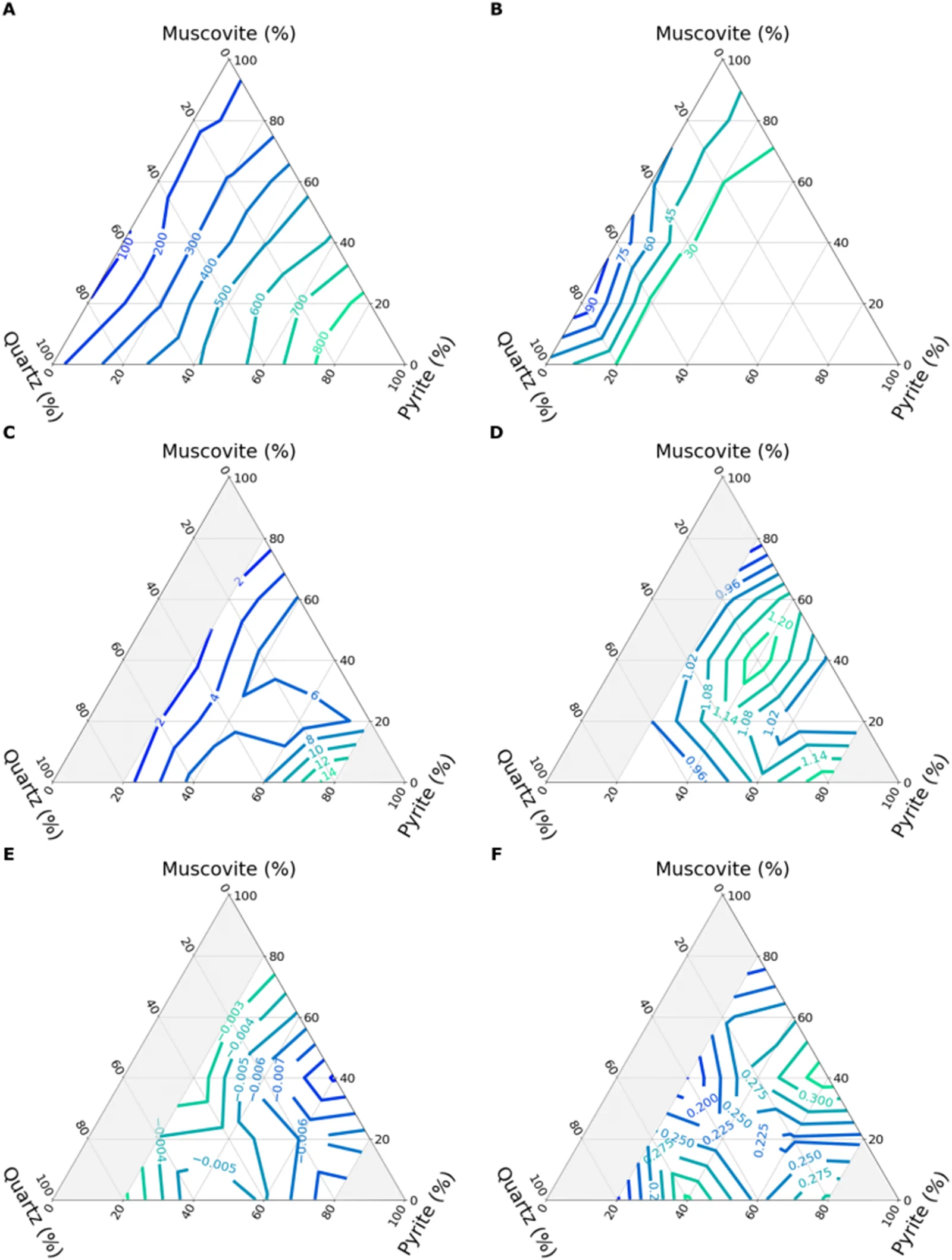
Ternary diagrams with contours for each electrochemical parameter. (A) Initial specific discharge capacity (mAh g^−1^) ternary diagram. (B) Capacity retention after 100 cycles at 1C (%) ternary diagram. (C) Parameter *a* of the power law fit for the pyritic peak values from the d*Q*/d*V* analysis. (D) Parameter *b* of the power law fit for the pyritic peak values from the d*Q*/d*V* analysis. (E) Parameter *c* of the linear fit for the silicate peak values from the d*Q*/d*V* analysis. (F) Parameter *d* of the linear fit for the silicate peak values from the d*Q*/d*V* analysis.

To further understand the impact of the pyritic and silicate components on capacity retention, differential capacity analysis was undertaken (methodology provided in SI S3.2) for the first 20 cycles of each ternary mixture. The peaks identified to the pyrite components of the capacity were modelled using a simple power law (eqn (1)), and the peaks identified to the silicate components of the capacity were modelled using linear regression (eqn (2)). The total capacitive contribution was modelled using eqn (3), a summation of the pyrite and silicate components. From eqn (3), parameters *a* and *d* correspond to the initial capacity contribution of the pyrite and silicates, respectively, while parameters *b* and *c* represent the rate of decay of capacity for the pyrite and silicate components, respectively.

1 Pyrite capacity component = *an*^−*b*^

2 Silicate capacity component = *cn* + *d*

3 Summed capacity component = *an*^−*b*^ + *cn* + *d*

Parameters *a*, *b*, *c*, and *d* were calculated for all ternary mixtures that contained both pyrite and at least one of quartz or muscovite and are shown in Fig. 4C, D, E and F, respectively. For the pyrite component parameter, *a* increases from 2 to 14 as pyrite content increases; however, parameter *b* shows the greatest values of >1.2 being at both the highest pyrite content and within the centre of the mixed regions. The trend in parameter *a* highlights the same trend seen in Fig. 4A; more pyrite increases the capacity component from the pyrite. The trend in parameter *b* is more complex, showing the capacity decay of the pyrite component to be most rapid when either in a purer mixture, as in Fig. 4B, but also in the most mixed systems, showing mixed systems to cause more rapid decay of the pyrite capacitive component. Parameter *d* shows a more complex relationship for the contribution of silicate minerals to the initial capacity, as shown in Fig. 4F. The values for *d* are lower with less pyrite and as a well-mixed ternary system in the centre of the plot. The values for *d* are greatest along the quartz-pyrite and muscovite-pyrite lines when they are at equal ratios. The values for parameter *c* in general increase in magnitude from −0.003 to −0.007 as pyrite content increases, as shown in Fig. 4E. The silicate component seems to decay more rapidly as pyrite content increases, but has the greatest initial capacitive components when in a binary equally split mixture with pyrite. The analysis of the differential capacity data shows that both the pyrite content and mixture system complexity, the more equal mixing of multiple phases without one mineral phase dominating the composition, impact the capacity values measured. The decay of the pyrite component, parameter *b*, is most rapid for both the pure pyrite and the more equally split ternary mixtures, showing pyrite decay to be negatively impacted by the complexity of the mixture, such as from a binary to a ternary mixture. This contrasts with the silicate component, where a simpler trend of increasing pyrite content leads to more rapid decay and larger negative *c* values. Parameter *a*, representing the initial pyrite component, increases with pyrite as expected. The trend in parameter *d*, representing the initial silicate component, is more complex, being greatest for the binary mixtures and the greatest pyrite contents. Increasing pyrite content improves the initial contribution of pyrite and silicates to the capacity while also leading to their more rapid decays. Mixture complexity is shown to lead to more rapid decays in the contribution of pyrite to capacity, but leads to greater contributions of silicates to the initial capacity values measured. Greater mineralogical complexity leads to a more complex electrochemical system, leading to greater variability in the performance of each mineral phase. Binary mixtures of pyrite with each silicate mineral will lead to more heterogeneous PSDs and particle morphologies, which can lead to improved packing arrangements of particles, increasing the wetting of particles by the electrolyte and leading to greater amounts of particle surface area exposure, enhancing silicate electrochemical activity.^44^

EIS measurements were taken pre-cycling and post-cycling, and fit to equivalent circuit models shown in Fig. S17 and S19. Initial *R*_ct_ values are shown to increase as silicate content increases in Fig. S18 from 95.89 Ω for pure pyrite to 172.4 Ω for pure quartz. This is due to the increased thermodynamic barrier for the lithiation of quartz compared to pyrite due to the requirement of breaking the Si–O bond.^55^ After cycling, the EIS spectra were fit to four impedance components (*R*_solution_, *R*_ct1_, *R*_ct2_ and *R*_ct3_) as shown in Fig. S20. The *R*_ct1_ and *R*_ct2_ components show gradual increases in impedance with increasing silicate content. A separate trend is observed with the *R*_ct3_ value, as the greatest impedance values are found within the centre of the ternary plot with the 20 : 40 : 40 pyrite : quartz : muscovite mixture exhibiting the greatest impedance of 4306 Ω. The lowest *R*_ct3_ values are seen for the pure quartz and muscovite electrodes, showing pure phase silicates to have lesser impedance values, due to them being good lithium-ion conductors.^61,62^

From the Warburg component of the EIS spectra collected pre- and post-cycling, the Warburg angle and *D*_Li^+^_ values were determined (methodology in Supplementary Material *S3.2*). Pre-cycling all Warburg angles are >60° inferring greater capacitive behaviour. Pre-cycling, *D*_Li^+^_ calculated using the Warburg region of EIS spectra exhibit similar *D*_Li^+^_ values to the equivalent values calculated for the pure mineral phases using GITT, with values in the range of 10^−14^ cm^2^ s^−1^ to 10^−15^ cm^2^ s^−1^. The agreement in *D*_Li^+^_ values pre-cycling between both methods highlights the reliability of determining *D*_Li^+^_ values from the Warburg component of EIS spectra for the mixed lithium-ion battery system tested. The subsequently calculated *D*_Li^+^_ values show a trend of the *D*_Li^+^_ value as smallest when silicate-rich (quartz and muscovite) and within equally mixed ternary mixtures. Post-cycling, all ternary mixtures which contained pyrite exhibit Warburg angles of 45° or less, while the quartz and muscovite mixtures continue to exhibit larger angles of >55°, inferring a continued capacitive behaviour in the silicate-only electrodes. The *D*_Li^+^_ values post-cycling exhibit decreased values; however, a new trend is observed with the quartz and muscovite silicate mixtures exhibiting lower *D*_Li^+^_ values. This trend is assigned to the difference in mineralogy, silicates compared to pyrite, and not the difference in particle size and PSD, as electrochemical kinetics are typically hindered by wider PSDs,^63^ but the silicate minerals have wider PSDs than the pyrite, as shown in Fig. 1A. This contradictory relationship between PSD and *D*_Li^+^_ shows electrochemical kinetics to be controlled mostly by mineralogy and not PSD width.

### Postmortem characterisation

To further understand trends in electrochemistry observed for the pure phases and ternary mixtures, *ex situ* characterisation of electrodes after the 100 cycles at 1C was undertaken (electrode preparation provided in SI S4). SEM images of post-cycled electrodes of pyrite, quartz and muscovite are shown in Fig. 5A, B and C, respectively. Corresponding EDX images are provided in Fig. S23–S25. Particle integrity is maintained for the pure phase quartz and muscovite electrodes with minimal electrode fracturing. For the post-cycled pyrite electrode, the pyrite particles are fractured into diminutive slabs, generating fractures at the electrode scale. This fracturing is expected as pyrite undergoes volume expansions of up to 159.2% during (de)lithiation.^49^ These same trends are seen in the 60 : 40 pyrite : quartz mixture in Fig. S26 and S27. Pyrite exhibits poor mechanical stability, leading to electrode scale fracturing, unlike quartz and muscovite, which exhibit good mechanical stability and minimal damage at the electrode level post-cycling.

**Fig. 5:**
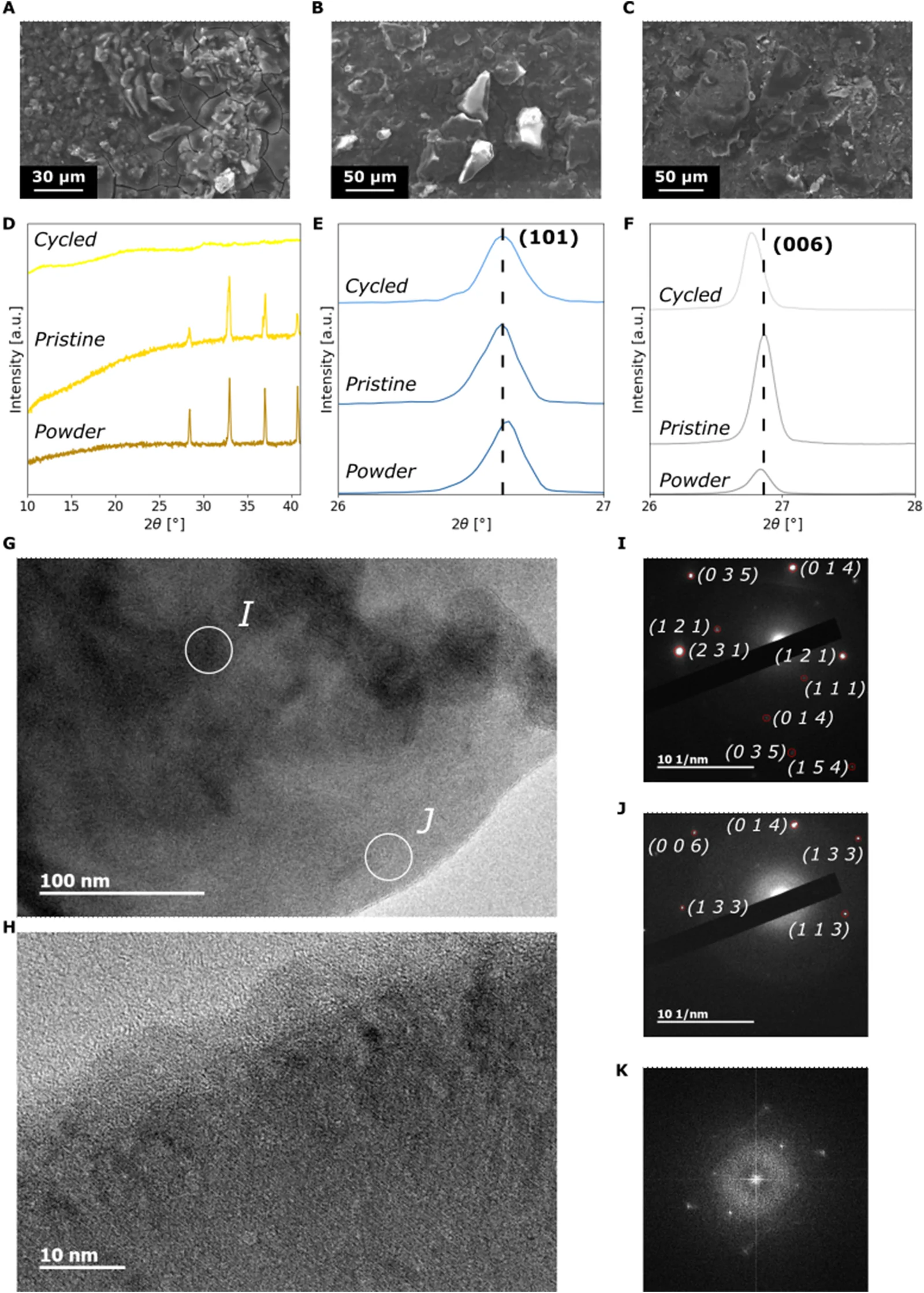
Postmortem analyses of electrodes after 100 cycles at 1C. (A) SEM image of cycled pyrite electrode. (B) SEM image of cycled quartz electrode. (C) SEM image of the cycled muscovite electrode. (D) ROI XRD patterns of pyrite electrodes before and after cycling. (E) ROI XRD patterns of quartz powder and quartz electrodes before and after cycling. (F) ROI XRD patterns of muscovite powder and muscovite electrodes before and after cycling. (G) TEM image of quartz particle post-cycling. (H) TEM image of pyrite particle edge post-cycling. (I) SAED image of region I. (J) SAED image of region J. (H) TEM image of the edge of the pyrite particle post-cycling. (K) FFT image of Figure (H).

To further probe the mechanistic processes impacting the trends in KPI during cycling, XRD measurements and TEM imagery were taken to understand the crystalline changes in the materials pre- and post-cycling. Region Of Interest (ROI) XRD patterns, highlighting finer pattern changes, are provided in Fig. 5D, E and F, respectively (Fig. S28A, B, and C provide the respective broad XRD patterns). Corresponding parameters from the Rietveld refinement are provided in SI S4. For the pyrite electrode, the increase in diffraction background intensity for all XRD patterns from 2*θ* values of 10° to 25° is due to the iron within the pyrite samples, as the Cu Kα sources excite the iron atoms, causing them to fluoresce.^64^ All peaks corresponding to pyrite are shown to disappear after cycling; pyrite has lost its crystallinity at the bulk level during cycling. Weak peaks at 30.0°, 32.0°, 33.6° and 36.9° for the cycled electrode are ascribed to Li_2_S, with broad peaks at 29–30°, 47–50° and ∼20° ascribed to the formation of other forms of iron sulphides such as marcasite and pyrrhotite. For the quartz electrode, peaks corresponding to quartz are maintained post-cycling with no shifting in their position, showing the crystallinity of quartz to be maintained. This is clearly shown in Fig. 4E by there being no 2*θ* shift in the characteristic (101) plane for quartz post-cycling. For the muscovite electrode, pre- and post-cycling five basal peaks are clearly noted: (002) at ∼8.9°, (004) at ∼17.7°, (006) at ∼26.8°, (008) at ∼36.0° and (0010) at ∼45.4°. The XRD signal from these planes is enhanced due to the preferred orientation of the muscovite flakes, as shown in Fig. 2D, lying flat on their basal plane, amplifying their basal plane signals. Fig. 5F reveals a clear shift to a lower 2*θ* value for the (006) basal plane, post-cycling, with a corresponding increase in the unit cell volume from 914 Å to 929 Å, likely due to the insertion of Li^+^ ions during cycling. Despite the lattice widening, the crystallinity at the bulk level is maintained, indicating lithiation to be limited. These XRD patterns show pyrite crystallinity to rapidly degrade, forming other iron sulphide phases, driving in part the rapid decay in capacity of the pure pyrite and pyrite-based mixture cells. Minor changes in the crystalline structure of quartz and muscovite during cycling show there to be minimal lithiation at the bulk scale for both silicate minerals. This corresponds with their lower-than-expected specific capacities, seen in Fig. 2A, highlighting the silicate minerals to be severely underutilised at the bulk scale.

To gain spatial information on the material's crystallinity at the particle level from micro to nano-scale, TEM imagery was undertaken on post-cycled electrodes of pyrite and quartz. Fig. 5G shows a TEM image of the edge of a micron-sized quartz particle after being cycled 100 times at 1C Select Area Electron Diffraction (SAED) images were taken near the edge (Fig. 5J) and the centre (Fig. 5I) of the particle. From the SAED images, it can be seen that the edge has been amorphized due to there being less clear diffraction spots, but also having a more prominent diffuse amorphous circle near the centre. The diffraction spots from the SAED image at the particle edge are weaker compared to the clear spots and lattice fringes seen in the pre-cycled electrode in Fig. S29 and S30. The quartz particles are shown to become slightly amorphous at the particle edge; however, it is not possible to discern whether amorphous silicon, likely formed by the partial reduction of the quartz silicate structures to silicon during the initial cycle, is present in addition to the presence of amorphous silicate. The lithiation of the quartz particles is hindered by its thermodynamic difficulty to lithiate^55^ leading to only the edges of particles lithiating due to their direct contact with lithium from the electrolyte. Fig. 5H represents the edge of a pyrite particle from a post-cycled electrode. Within an amorphous background, clear lattice fringes with a width of 0.180 nm are noted and correspond to the (200) lattice plane of FeS (marcasite), a known product of pyrite (de)lithiation. The FFT image of Fig. 5H is shown in Fig. 5K, showing clear crystallinity within the sample with a non-cubic arrangement of bright spots like that of marcasite and not pyrite. Pyrite is shown to lose crystallinity at a bulk level but maintain it at a nano level in the form of marcasite. This loss of crystallinity at the bulk level corresponds with the capacitive degradation of pyrite during discharge, being driven by decreases in the intercalation plateau at ∼2.0 V of the (dis)charge curves of Fig. S7A and D, as the crystalline structure for the Li^+^ ions to intercalate into is lost during cycling. In addition, Fig. S30 and S31 show the formation of nano Li_2_S particles ∼5 nm in size with lattice spacings of 0.327 nm, indicative of the (111) plane for Li_2_S. These nanoparticles are shown to preferably form on the super p carbon nanoparticles, as the STEM image and EDX spectra in Fig. S33 show the carbon-rich regions to contain greater amounts of sulphur and have a sharp spike in counts at exceptionally low energy levels, suggestive of lithium. The main products of the lithiation of the pyrite are amorphous regions with nano regions of marcasite, with the formation of nano lithium sulphide particles preferentially on the conductive super p carbon particles of the electrode. These postmortem analyses provide direct mechanistic insights into the capacitive behaviour of each pure mineral phase. XRD patterns reveal minimal or no change in silicate minerals, quartz, and muscovite, crystallinity during cycling, and minimal changes in particle integrity are observed from SEM images, post-cycling. These analyses show these natural silicate minerals to undergo minimal lithiation during cycling, causing their measured capacities to be less than their theoretical capacity of 1965 mAh g^−1^. In contrast, pyrite is shown to lose crystallinity at the bulk level during cycling, with crystallinity only maintained at the local scale, with marcasite forming at the nanoscale. This loss in crystallinity, coupled with the particle and electrode damage seen in the SEM-EDX images, is a direct cause of the rapid decay in pyrite capacity during cycling.

## Discussion

### Pure mineral phases

Differing trends are observed for pyrite and the two silicate minerals, quartz and muscovite. Both quartz and muscovite exhibit similar performance in terms of initial discharge capacity and capacity retention, despite their difference in particle size, shape and morphology, highlighting the differences in electrochemical performance among the pure phase minerals to be mainly controlled by the difference in mineralogy: pyrite and silicates. Analysis of EIS spectra shows differing trends, with the *R*_ct_ for quartz increasing and for muscovite gradually decreasing. XRD patterns show the muscovite crystal lattice to expand when accommodating Li^+^ ions, allowing for the easier diffusion of Li^+^ ions during cycling and reducing the *R*_ct_ during cycling. The silicate minerals exhibit specific capacities of ∼100 mAh g^−1^, much lower than the theoretical specific capacity of silica of 1965 mAh g^−1^. This is due to minimal levels of lithiation at the bulk level; the bulk crystallinity of quartz does not change, with only the partial lithiation of particle edges, generating amorphous SiO_2_ structures, increasing *R*_ct_ values during cycling. Lithiation of both silicates is hindered by the high thermodynamic energy barrier, leading to increased *D*_Li^+^_ coefficients post-cycling for both silicate minerals. Pyrite, as in the literature, exhibited vastly different electrochemical behaviour. Initial discharge capacities were high; however, capacity retention was poor. Unlike the silicates, the pyrite is shown to lose its crystallinity and particle integrity at the bulk level, forming nanoparticles and regions of Li_2_S and marcasite, rapidly reducing the capacity of the pyrite from intercalation mechanisms during cycling as seen in the (dis)charge curves.

### Mixing trends

Mixing of the three mineral phases did not generate consistent trends in the different electrochemical parameters measured. The impact of varying ratios of quartz and muscovite had a negligible effect on KPIs, as both silicates exhibit a similar response despite their differences in particle size, distribution and morphology, showing these factors to have minimal impact on the trends in KPIs discussed. Initial specific discharge capacity was shown to linearly increase as pyrite composition increased; however, the addition of pyrite to any mixture led to a rapid drop in capacity retention. From differential capacity analyses, increasing pyrite content is shown to lead to more rapid decays in the pyrite and silicate component to capacity. Mixture complexity is shown to cause more rapid decays in the pyrite component to capacity, while in contrast leading to greater initial components of silicate to capacity when within binary mixtures. Mixing is also shown to generate greater *R*_ct_ values and *D*_Li^+^_ values after cycling, negatively impacting performance. Both mineral type and complexity of mixture are shown to impact all electrochemical KPIs, with the trends different between parameters and not just a function of the weighted average of the minerals present within the mixture.

These trends highlight the adverse effect of mixing active materials for this ternary system of natural minerals studied. Depending on the end use, either a highly pure pyrite phase or a highly pure silicate phase should be considered. Pyrite has high initial specific discharge capacity but poor capacity retention and *D*_Li^+^_ values post-cycling. Its main application could be as a primary battery or a low cycle number but high energy density battery. Silicate minerals with low concentrations of pyrite exhibit low initial specific discharge capacity but good capacity retention and *D*_Li^+^_ values after cycling; potential future applications could be for low-energy but long-term applications,^65^ however, all battery KPIs must be considered, such as weight, cost, form factor, safety and thermal properties, in addition to the electrochemical performance metrics outlined before decisions on viability are made.^66^ Mixed mineral sources as an active material for batteries are viable; however, the end use must always be considered, as compromising certain KPIs could lead to a battery with no defined end-use. Potential avenues to improve electrochemical performance for real-world applications could include the use of purification strategies used for lithium-ion battery recycling: pyrometallurgical,^67^ hydrometallurgical^68^ or physical separation^69^ technologies. In addition, material enhancement strategies could be considered, such as particle nano-sizing and conductive additive coating, which have been previously used to improve the performance of pyrite^10,45,53,70–72^ and quartz^55,60,73,74^ within lithium-ion batteries. However, the potential benefits of these strategies must be weighed against their cost, both financially and environmentally, including increased energy and water use, to adequately assess their potential applicability.

To judge the effectiveness of this ternary system, five KPIs were compared for the three minerals studied and two mine tailings,^35^ acting as natural mineral sources, as shown in Fig. 6. Silicate minerals are shown to be robust with excellent rate performance, *D*_Li^+^_ values and capacity retention, while pyrite has the best initial specific discharge capacity and *R*_ct_ values. The High Pyrite (HP) tailings, with 98.84% pyrite and pyrite mixes and <1% quartz (composition in Table S4), cover the smallest area of the radar graph in Fig. 6. The introduction of impure phases to the HP tailings leads to worsened performance in two out of five of the KPIs when compared to pyrite. In contrast, the LP tailings with only 16.03 wt% pyrite and 45 wt% quartz and orthoclase feldspar (composition in Table S5) outperformed the individual silicates in terms of initial impedance, *D*_Li^+^_ and initial specific discharge capacity with equal rate performance, and lower capacity retention. The introduction of pyrite as a low wt% impurity to a silicate-dominant mixture shows general improvements in electrochemical performance. The introduction of minor mineral phases to a dominant mineral phase has different impacts on the dominant mineral phase's electrochemical performance. The mine tailings show the benefits of pyrite diminish with the addition of minor mineral phases, while the benefits of silicates are shown to be enhanced with the addition of pyrite as a minor mineral phase when compared with the KPIs of the pure mineral phases. When compared to the 21 ternary mixtures, this same trend is observed for capacity retention, as any additions of pyrite >20 wt% are shown to rapidly diminish capacity retention. However, for other electrochemical performance metrics such as initial discharge capacity, the trend shown in Fig. 6 is not directly observed.

**Fig. 6:**
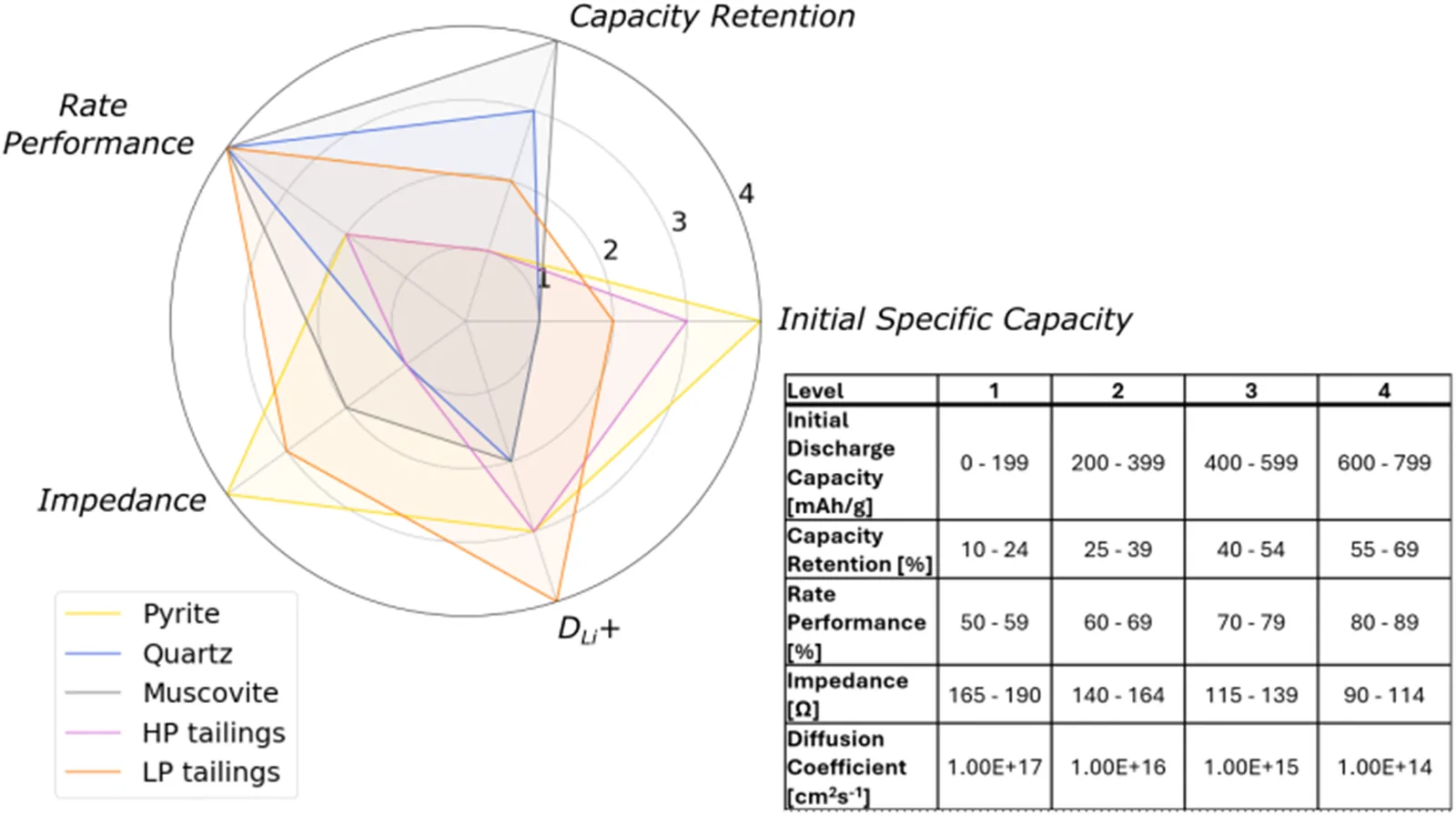
Radar graph highlighting the KPI of the pure phase minerals with mine tailings^35^ containing these minerals.

## Conclusion

This work provides an in-depth study of the impact of mixing natural minerals as active materials within battery electrodes on electrochemical performance. To secure future supply chains for battery materials and increase their sustainability, direct use of mixtures of natural minerals is a viable option. Mine tailings are an example of a natural mineral source which can be directly used within battery electrodes, as it contains an abundance of electrochemically active minerals for Li-ion batteries. Benefits include reduced environmental impacts and promotion of circular economy ideals, crucial to sustainable development. To characterise these complex natural mineral systems, a simplified ternary system was designed containing pyrite, synonymous with sulphide mine tailings and thoroughly researched in battery literature, quartz, the most abundant mineral from the literature review and muscovite, the third most abundant mineral from the literature review with a different phyllosilicate structure compared to the tectosilicate structure of quartz. This synthetic system allowed for a systematic approach to identifying the impact of different minerals within a mixture on electrochemical performance without the highly variable complexity of using mine tailings.

Studies of the pure phase minerals show both silicates, quartz, and muscovite, to behave similarly electrochemically. Pyrite acts electrochemically differently, showing greater initial performance with more rapid capacity decay. These trends are linked to the loss of bulk crystallinity and particle fracturing. Particle integrity is maintained for both silicates with limited changes in crystallinity post-cycling. Consistent trends between mineral type and mixture complexity are shown for the KPIs studied. Initial specific discharge capacity linearly increased with pyrite content; however, capacity retention reduces dramatically after the inclusion of >20 wt% pyrite, creating a trade-off between initial capacity and battery longevity. Post-cycling, *D*_Li^+^_ and *R*_ct_ values are shown to decrease the most within equally mixed mixtures. In addition, the pyrite component of capacity from differential capacity analyses is shown to decay more rapidly when in equally mixed mixtures. Comparison of the tri-phase mixture performances with real mine tailings shows the introduction of minor mineral phases to impact the electrochemical performance of the major mineral phase differently depending on the major mineral phase present; silicate mineral electrochemical performance is enhanced with the addition of small quantities of pyrite, while pyrite electrochemical performance is reduced with the introduction of small quantities of silicates.

This work highlights the trade-off between different electrochemical KPIs for naturally derived mixed mineral systems. The end use of a battery must be considered when deciding if a mixture is acceptable: for example, for low energy with performance longevity or high energy short longevity uses. Enhancement strategies, including mineral purification and materials-based enhancements, can be used to tailor the mixed mineral systems for industry applications; however, decisions must be made with their additional economic and environmental costs in mind. With this approach, trade-offs can be evaluated for natural mineral sources, allowing them to be used effectively and sustainably as battery materials.

## Author contributions

Conceptualisation, JDM, KH, CG and PRB; methodology, JDM; investigation, JDM; resources, KH, CG and PRB; writing – original draft, JDM; writing – review and editing, JDM, KH, CG and PRB; visualisation, JDM; supervision, KH, CG and PRB.

## Conflicts of interest

The authors declare no competing interests.

## Supplementary Material

EB-002-D6EB00037A-s001

EB-002-D6EB00037A-s002

EB-002-D6EB00037A-s003

EB-002-D6EB00037A-s004

EB-002-D6EB00037A-s005

## Data Availability

All data supporting the findings in this study are available within the paper, supplementary information (SI) and three attached workbooks (W1, W2 and W3). Workbook W1 contains the fully cited mine tailings compositions, workbook W2 contains the geographical location of all mine tailings compositions cited and workbook W3 contains all the electrochemical results for the mixtures presented in this article as raw data in a tabulated form. Supplementary information is available. See DOI: https://doi.org/10.1039/d6eb00037a.
