# Supplementary material for: Electrochemical variability of natural mineral mixtures as electrode materials in Li-ion batteries
Source: EES Batter. 2026 Jul 21;2(5):1592–607. doi: 10.1039/d6eb00037a (PMC13655888; doi:10.1039/d6eb00037a)
Supplement: Supplementary file 1 [file EB-002-D6EB00037A-s002.pdf]

Supplementary Information (SI) for EES Batteries.
This journal is © The Royal Society of Chemistry 2026

Supplementary Material

S1 Mine Tailings Clustering Analysis Dendrogram

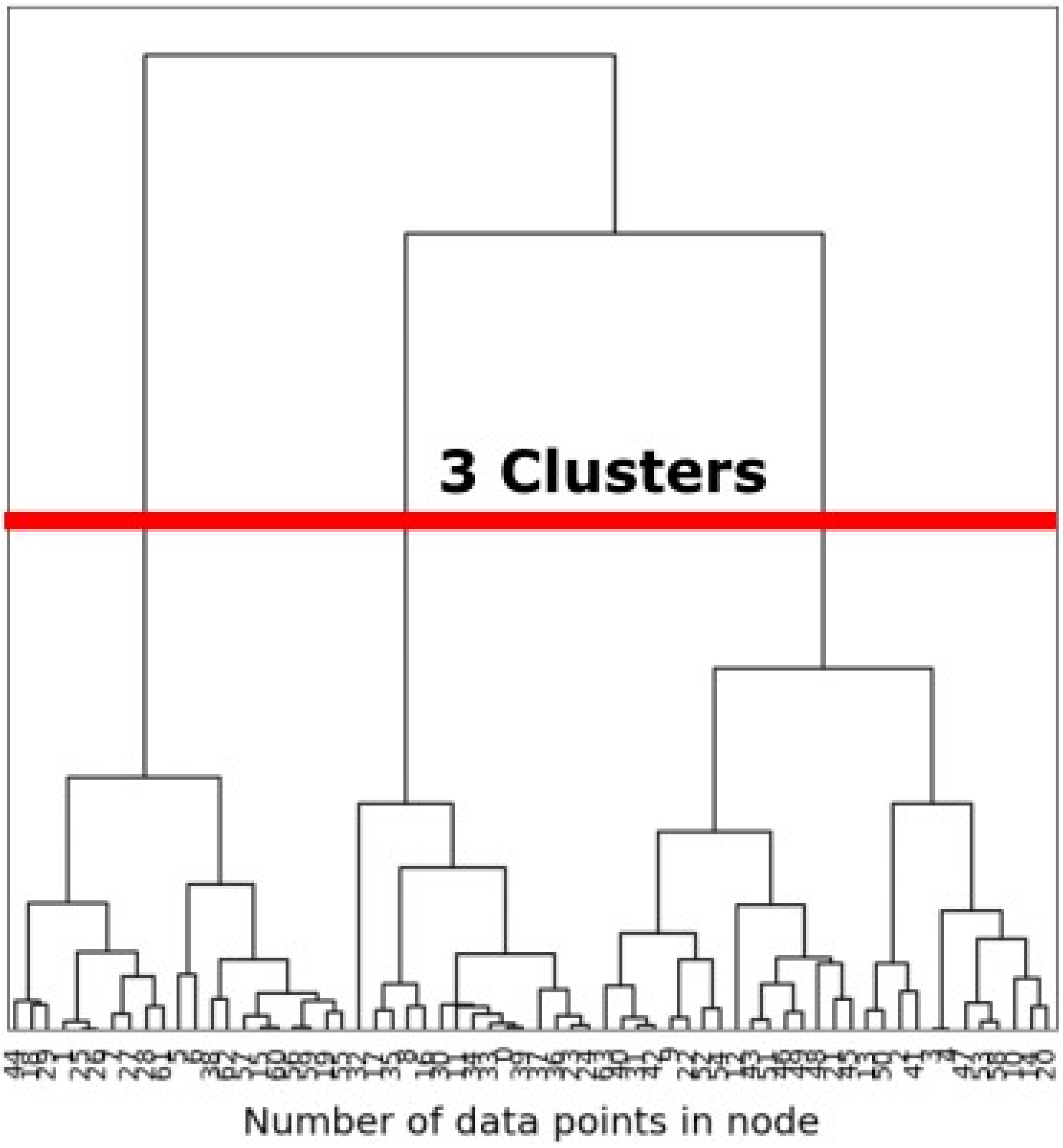

Figure S1: Dendrogram of the hierarchical clustering of the mine tailings compositions.

S2 Pre-Cycling SEM and EDX Elemental Maps

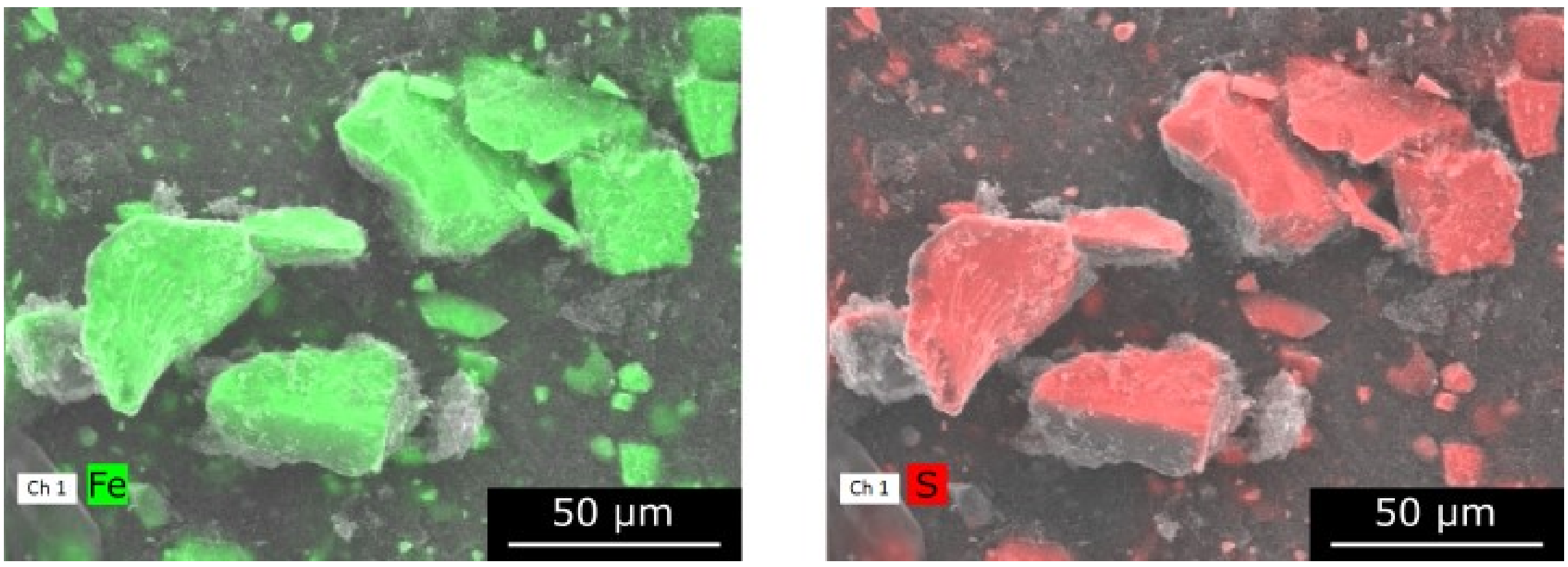

Figure S2: EDX maps for Fe and S for a pyrite electrode before cycling.

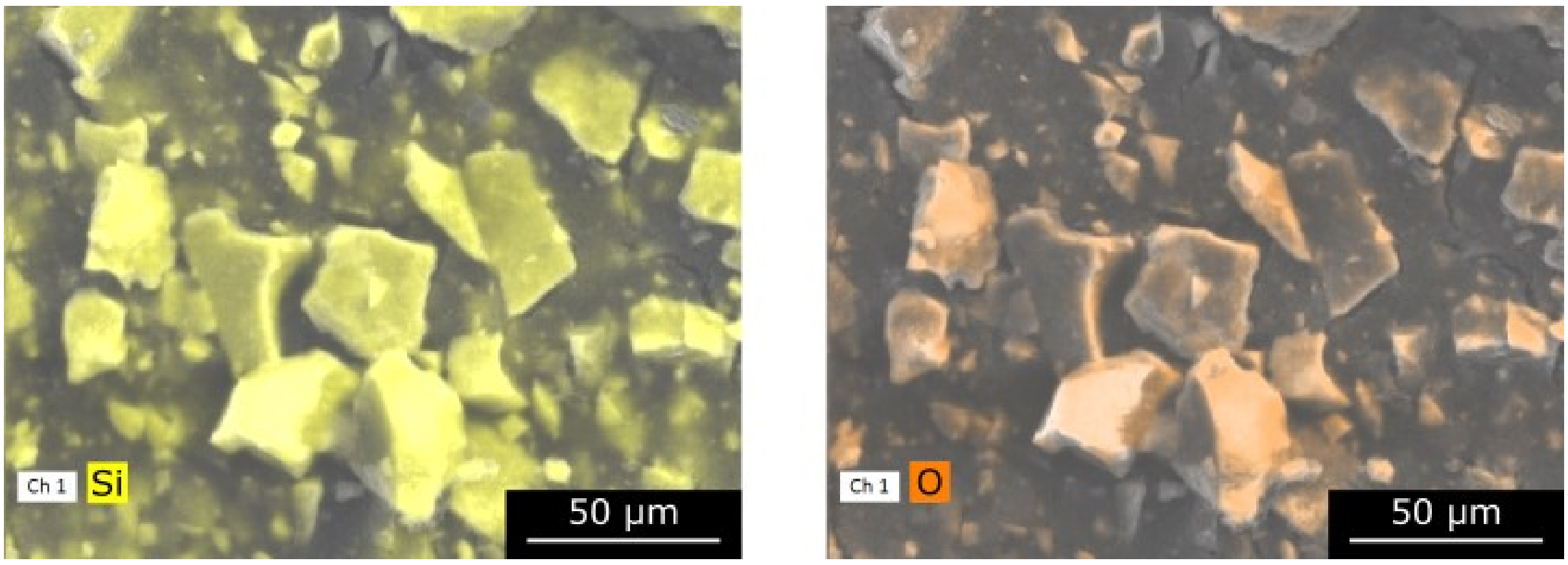

Figure S3: EDX maps for Si and O for a quartz electrode before cycling.

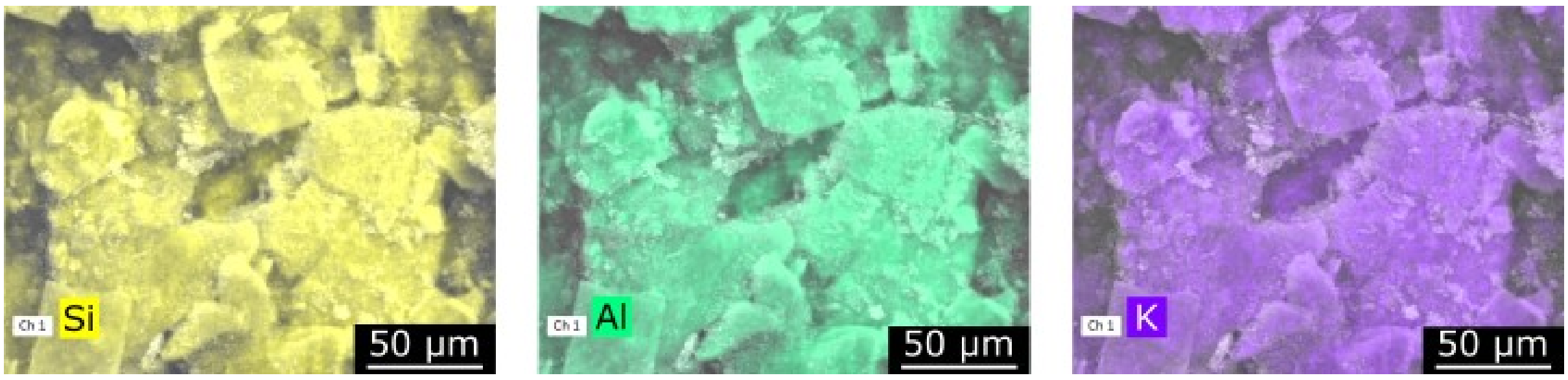

Figure S4: EDX maps for Si, Al, and K for a muscovite electrode before cycling.

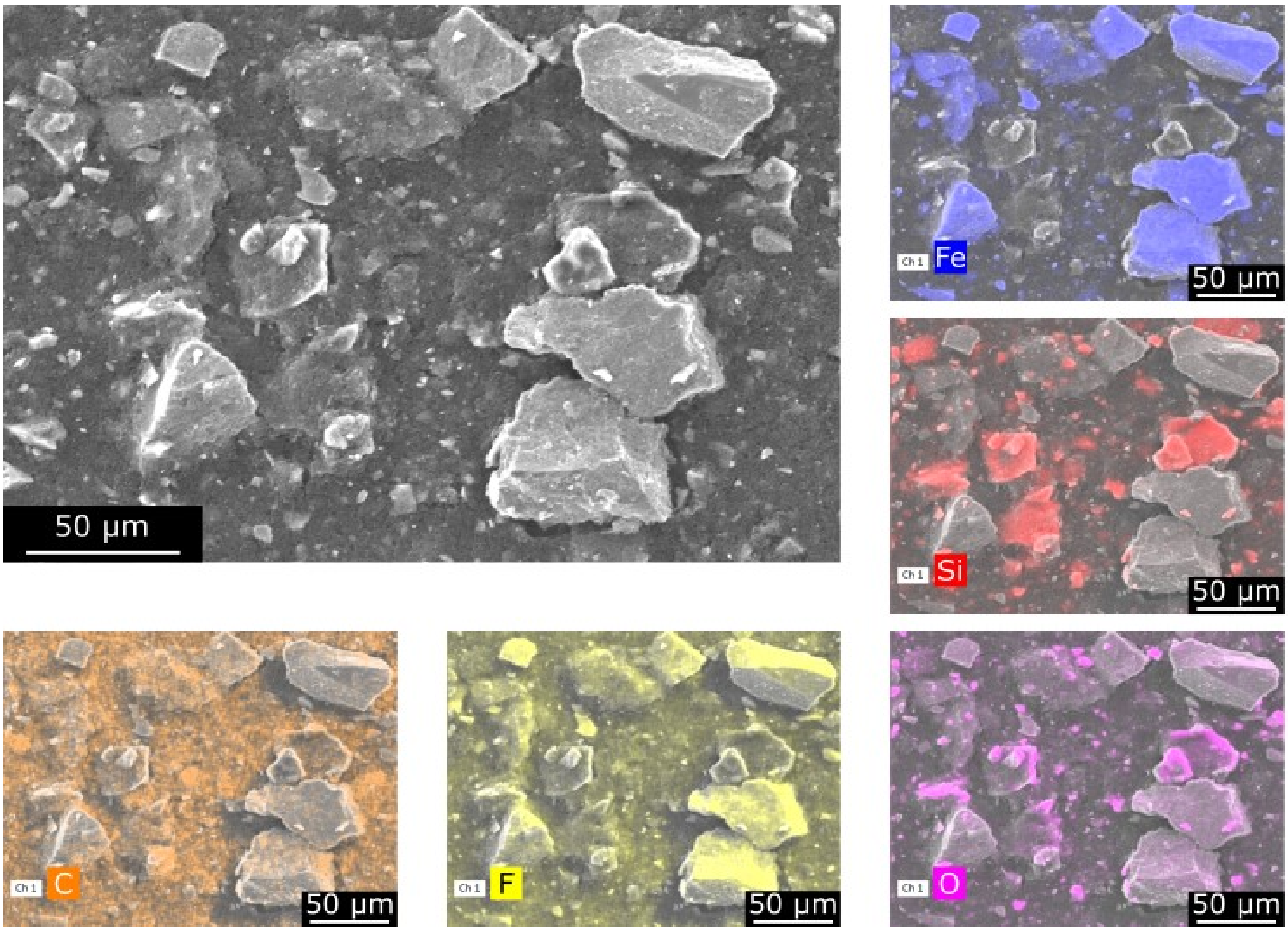

Figure S5: SEM image and EDX map for a 60% pyrite and 40% quartz electrode pre-cycling. Elemental maps are shown for Fe, Si, C, F and O.

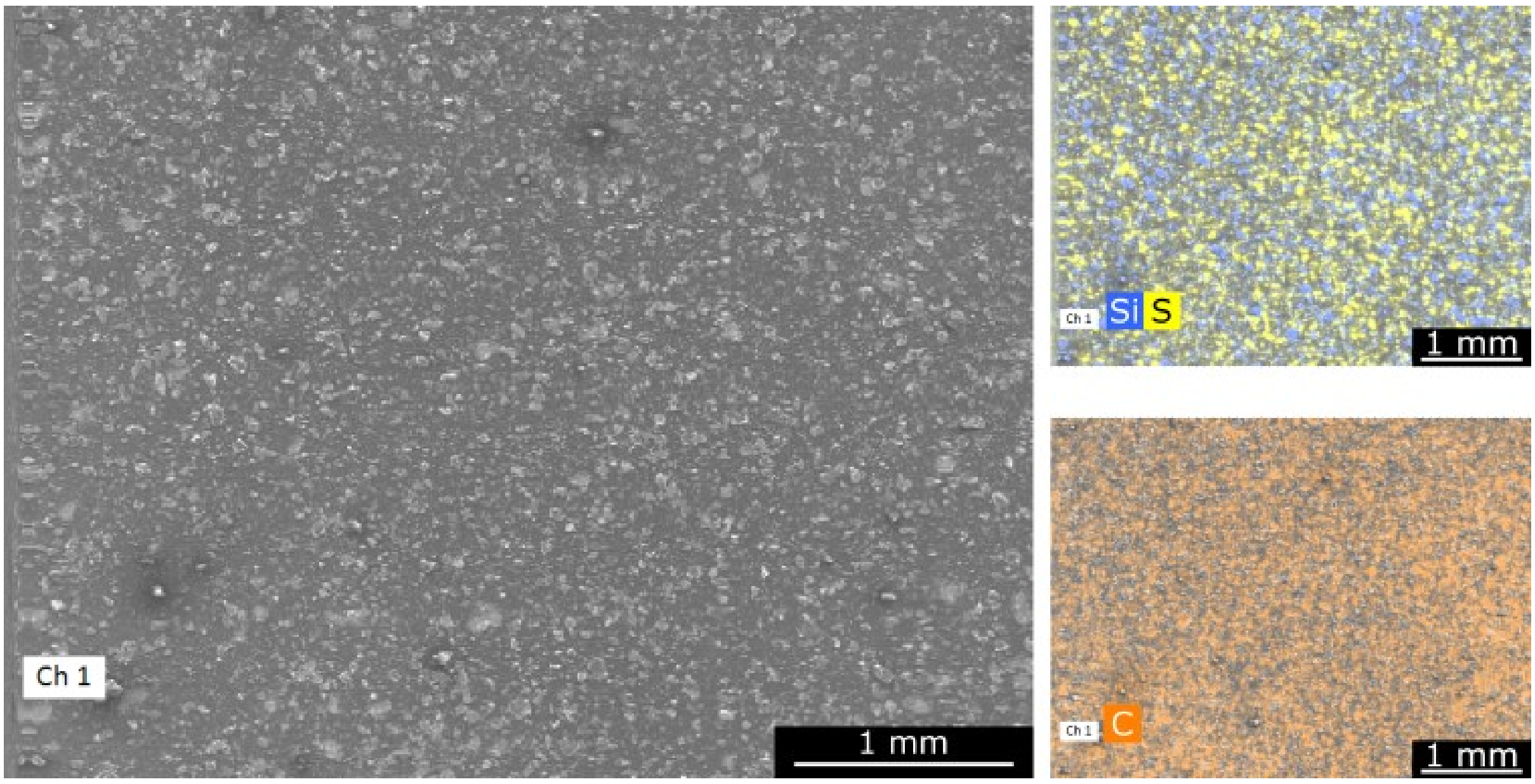

Figure S6: SEM image and EDX maps of a 60% pyrite and 40% quartz electrode pre-cycling for the elements Si, S and C.

S3: Further Electrochemical Testing

S3.1: Pure Phase Minerals

Charge/Discharge Curves

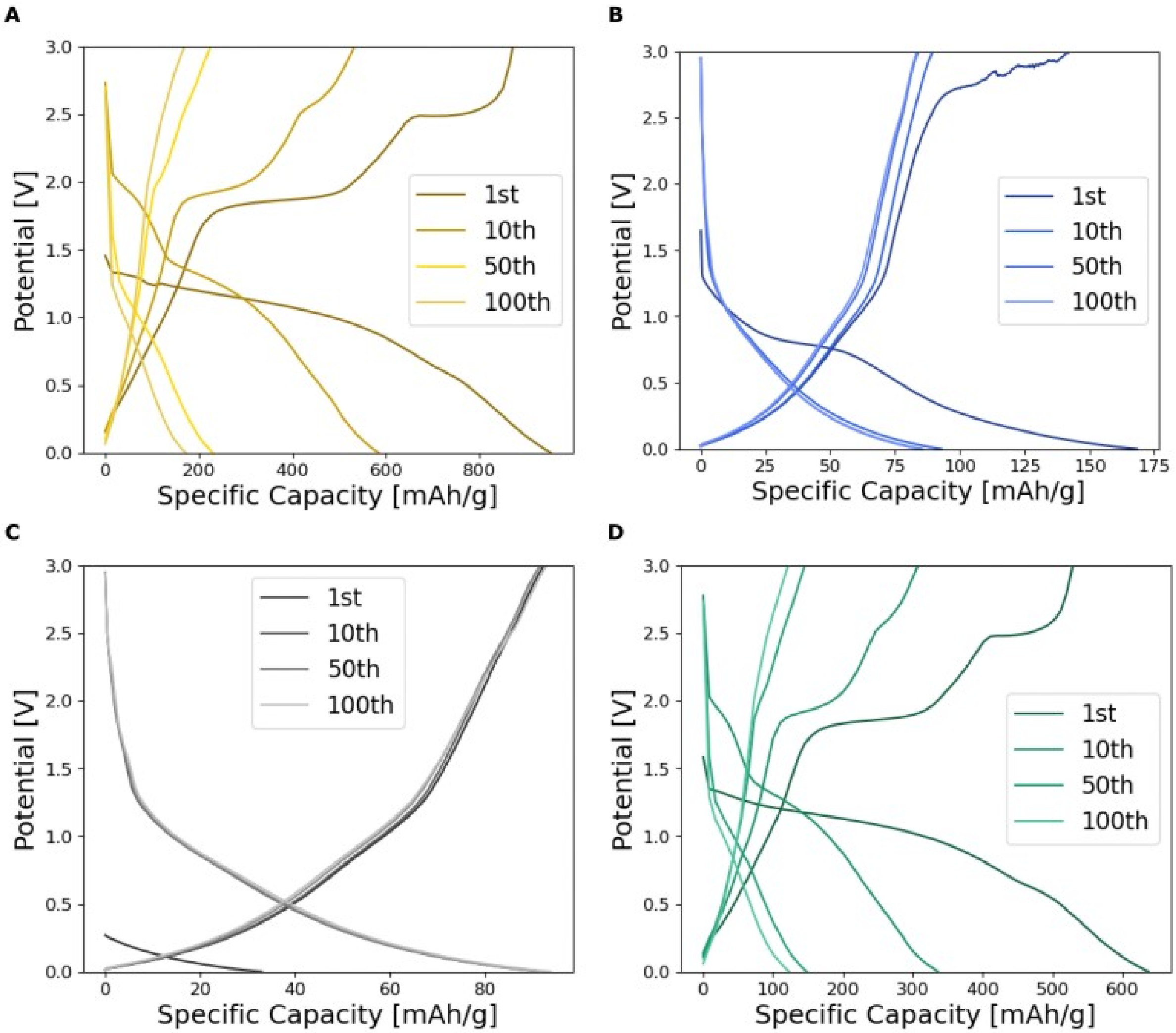

Figure S7: Galvanostatic charge/discharge curves of cycles 1, 10, 50 and 100 for the pure mineral phases and example mixture. A) Pyrite. B) Quartz. C) Muscovite. D) 60 % pyrite and 40 % quartz.

Mixture CV

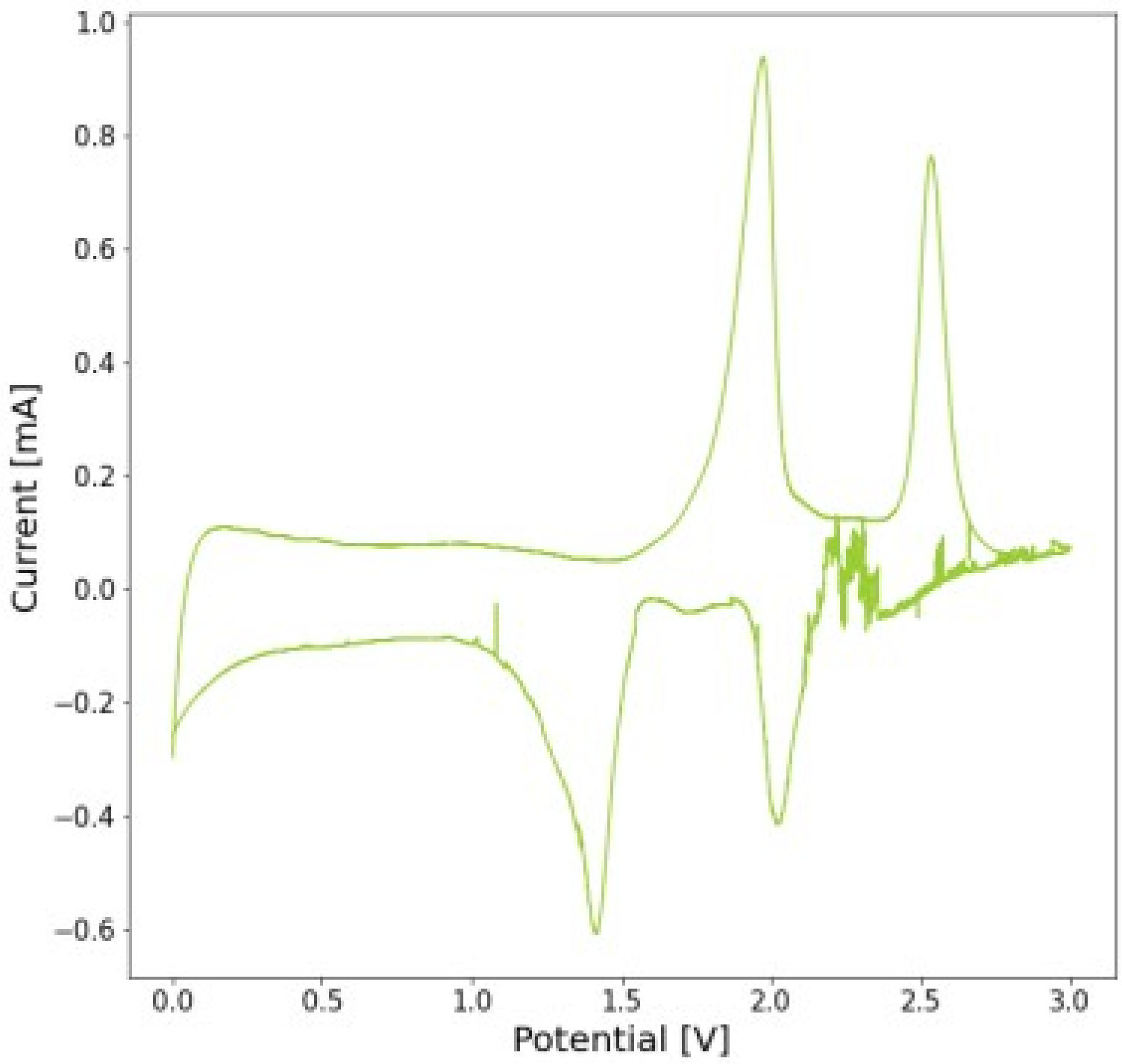

Figure S8: Initial CV cycle of a 60% pyrite and 40% quartz electrode with a 0.1 mV/s scan rate.

0.1C Cycling Performance

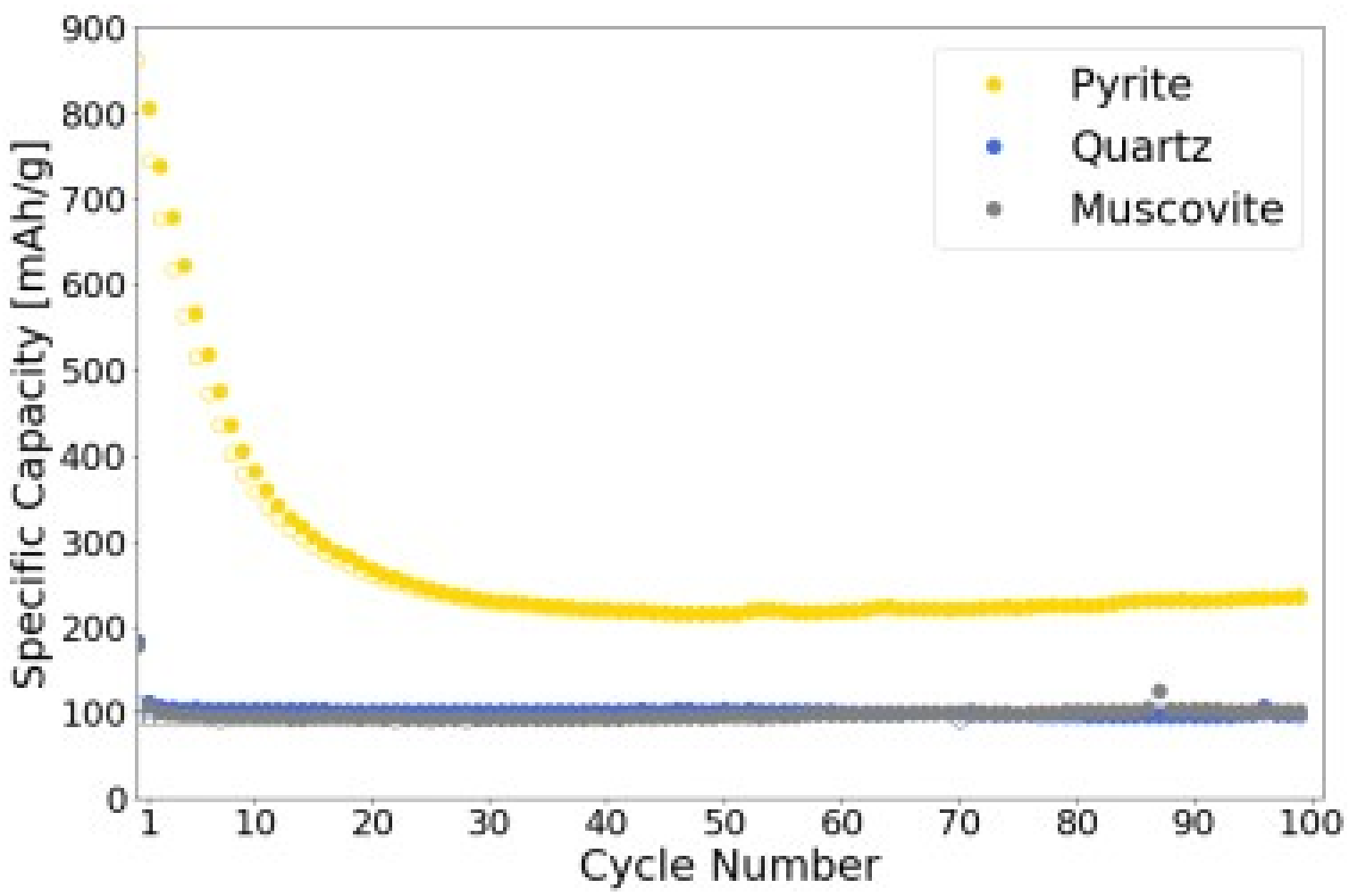

Figure S9: Capacity against cycle number plot for pyrite, quartz and muscovite cycled at 0.1 C.

EIS Spectra and equivalent circuits

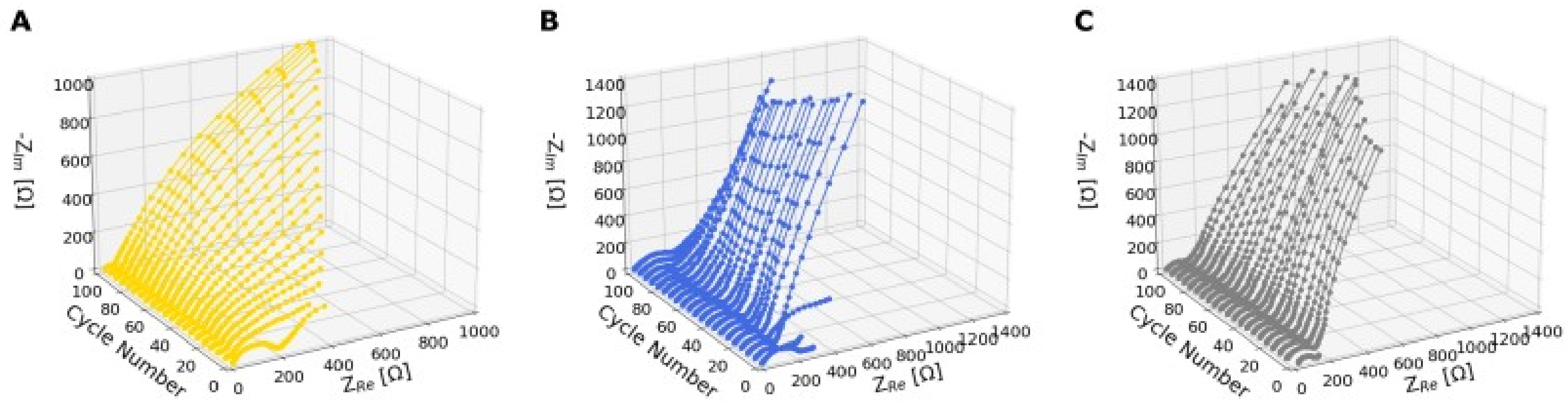

Figure S10: EIS spectra, after every 5 cycles when cycled at 1C, for pure phase mineral electrodes. A) pyrite, B) quartz and C) muscovite.

D_{Li+} Determination using GITT

The Lithium-ion diffusion coefficient (D_{Li+}) was calculated using Equation S1:

$$D_{Li^+} = \frac{4}{\pi\tau}\left(\frac{m_b V_m}{M_b S}\right)^2\left(\frac{\Delta E_s}{\Delta E_t}\right)^2$$

(S1)

τ is the time of the current pulse, m_b is the mass of active material within the electrode, V_m is the molar mass of the active material, M_b is the molecular mass of the active material, S is the electrode area, and ΔE_s is the steady state potential, and ΔE_t is the potential change during the current pulse once the iR drop is removed.

The values used for parameters chosen for each pure mineral phase are shown in Table S1. The time for the current pulse, τ, was chosen as 420 s (7 minutes) as the relationship between the potential and the square root of the time was determined to be linear, an assumption made during the formulation of Equation S1.

Table S1: Table showing the parameters used for the calculation of D_{Li+} for both tailings samples from the GITT data collected.

**Parameter**	**Pyrite**	**Quartz**	**Muscovite**
**V_m [cm^3mol^{-1}]**	23.942	22.69	140.55
**m_b [g]**	0.00093562	0.00106806	0.001169
**M_b [$gmol^{-1}$]**	120.082	60.085	398.326
**T [s]**	420	420	420
**S [cm^2]**	$7.0^2\pi$	$7.0^2\pi$	$7.0^2\pi$
**I [A]**	0.00084 (1C)	0.0001 (1C)	0.00012 (1C)

The overpotential (η) values were determined from the GITT analysis as the potential difference between the end of the relaxation time after the current pulse and the start of the relaxation time after the current pulse. The internal resistance was then determined using Equation S2:

$$Internal\ Resistance\ [\Omega] = \frac{\eta}{|I_{Applied}|}$$

(S2)

Figures S11, S12 and S13 provide the overpotential, internal resistance and D_{Li+} values for the first discharge and charge for pyrite, quartz, and muscovite, respectively.

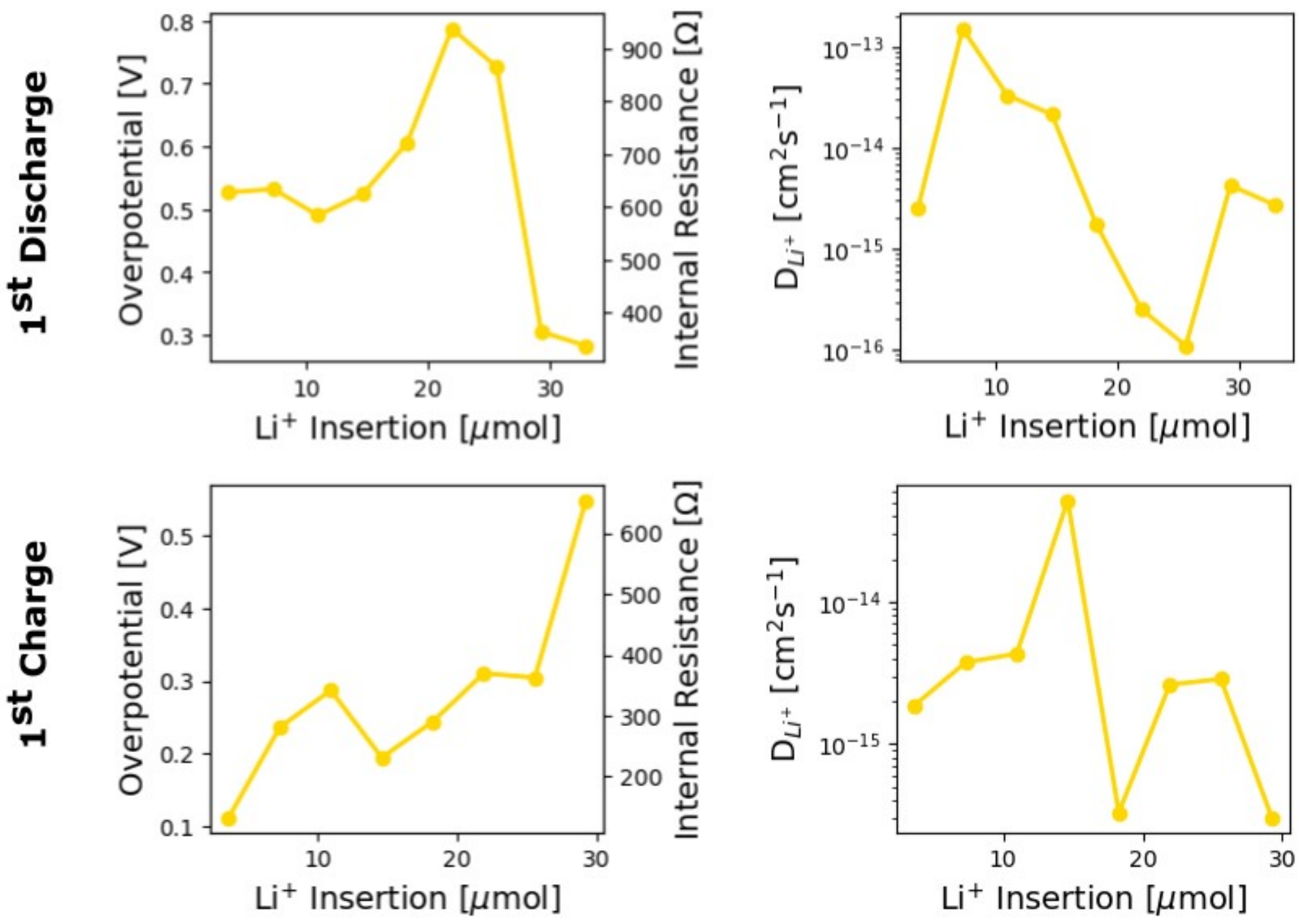

Figure S11: Plots of overpotential, internal resistance and D_{Li+} against Li^+ insertion for the first charge and discharge of a pure pyrite electrode.

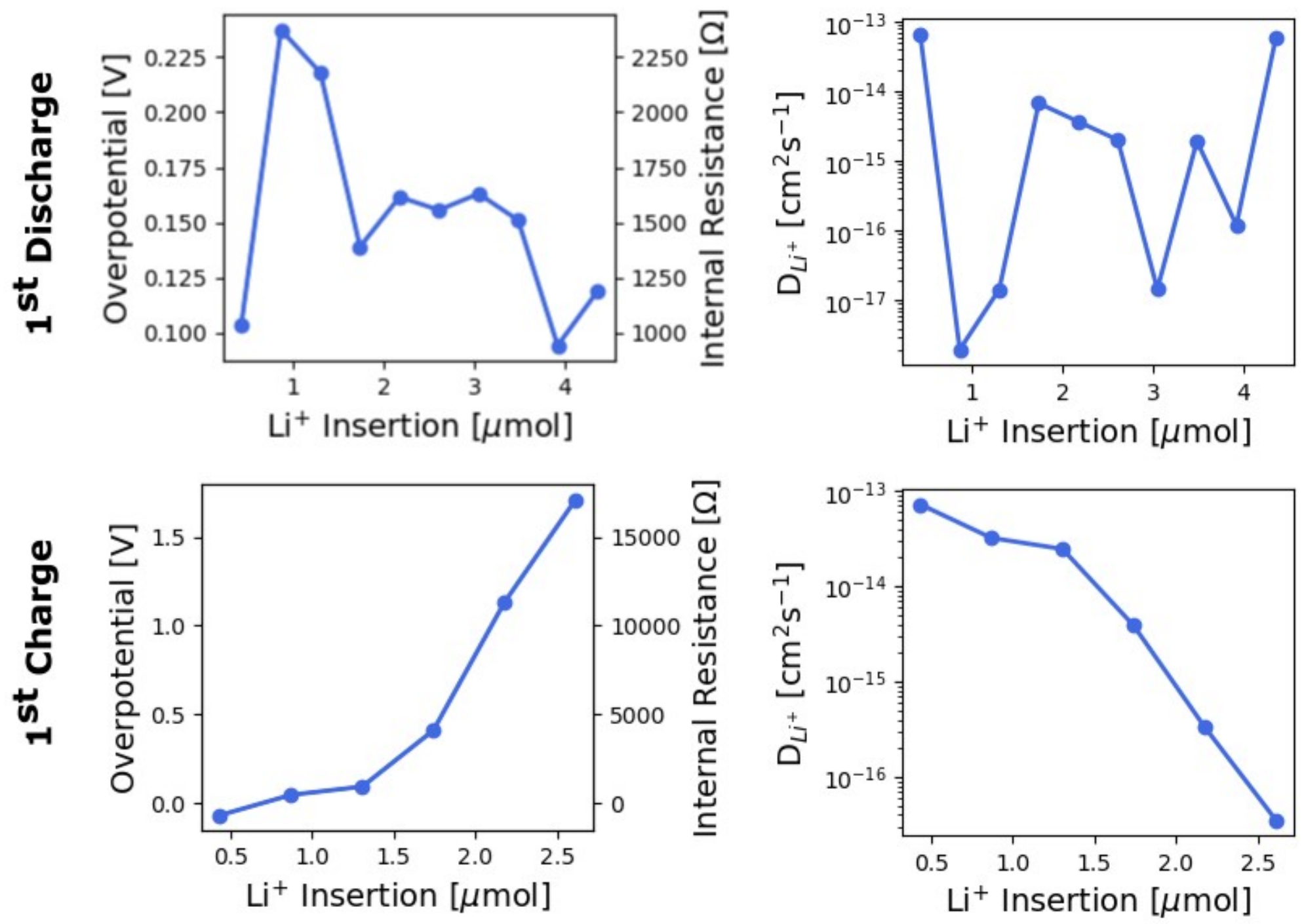

Figure S12: Plots of overpotential, internal resistance and D_{Li+} against Li^+ insertion for the first charge and discharge of a pure quartz electrode.

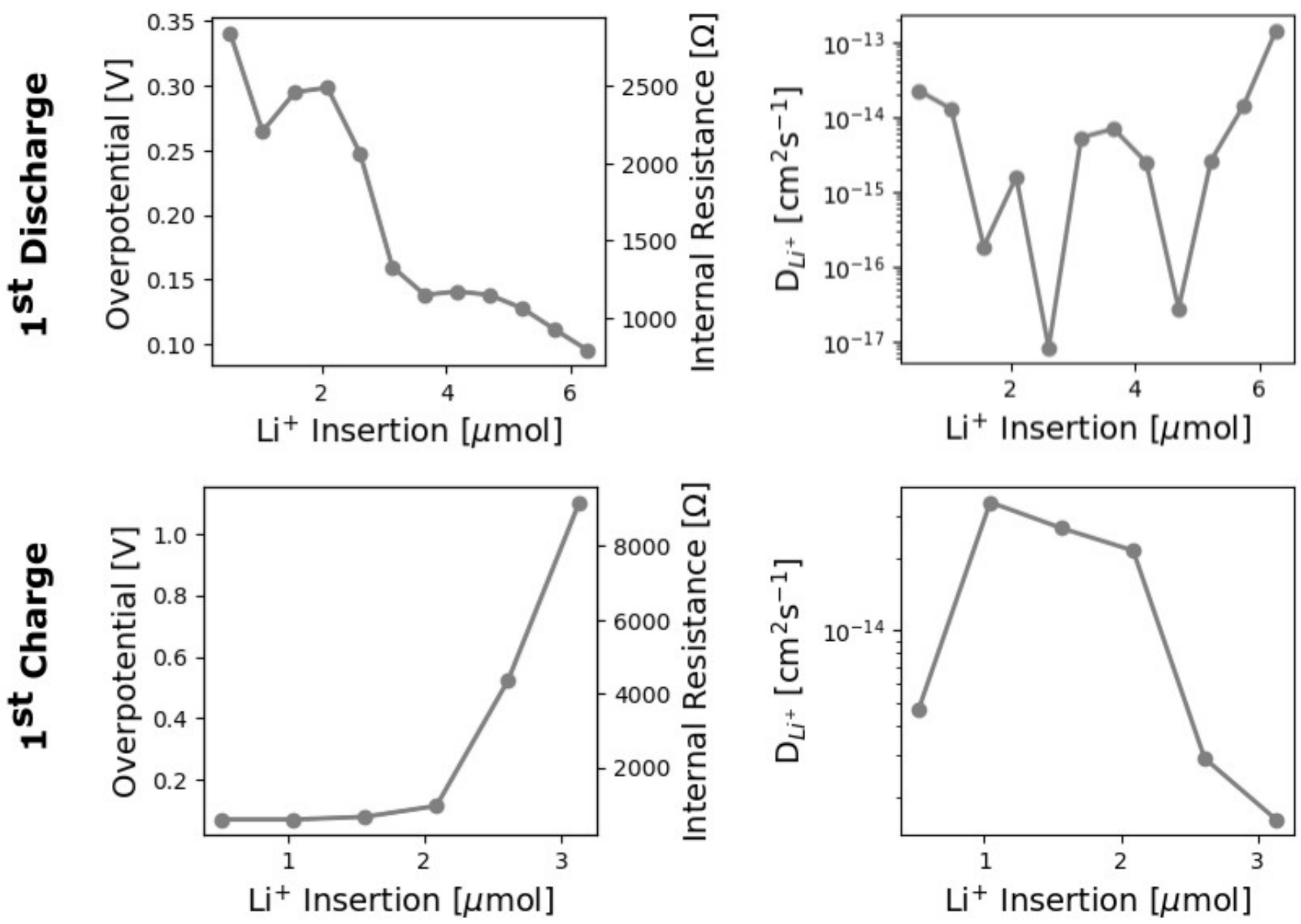

Figure S13: Plots of overpotential, internal resistance and D_{Li+} against Li^+ insertion for the first charge and discharge of a pure muscovite electrode.

3.2: Ternary Mixtures

Differential Capacity Analysis

The differential capacity analysis was undertaken on the first 20 cycles of each ternary mixture anode, as shown in Figure S14A; the peaks were hard to identify after 20 cycles. The peaks associated with pyrite and silicates, highlighted in Figure S14A for the 80% muscovite and 20% pyrite electrode, were summed and their relative components plotted in Figure S14B. From these data values, Equations 1and 2 were fit to the differential capacity components for pyrite and silicate. The red dotted line in Figure S14B highlights the adequate fit of the summed components to this model.

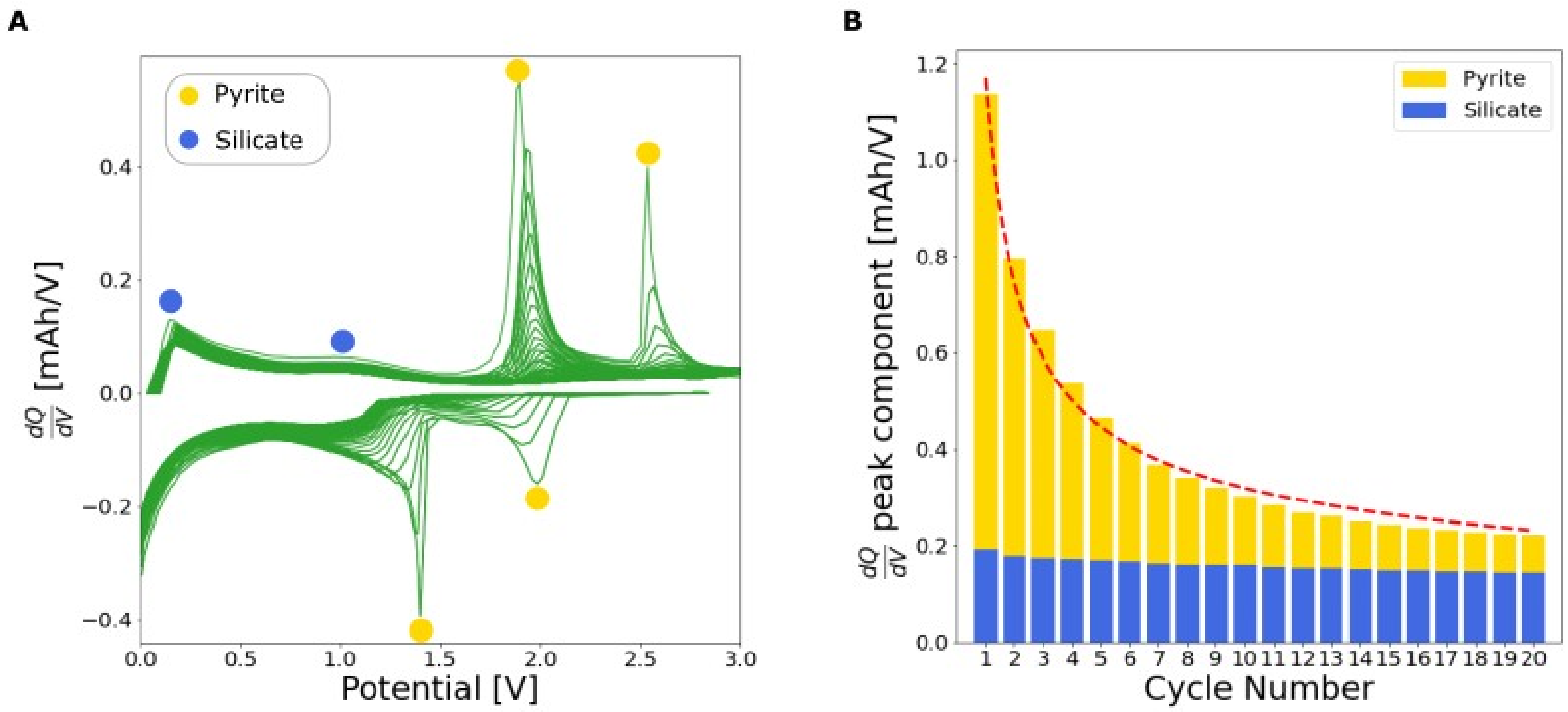

Figure S14: Example dQ/dV plots for the 20% pyrite and 80% muscovite electrode. A) dQ/dV plot. B) Bar chart of pyrite and silicate component of the first 20 cycles from the dQ/dV plot. The red dashed lines represent the fitted model from Equation 3.

The r^2 value was determined for each ternary mixture analysed, and the ternary diagrams in Figure S15 for the pyrite and silicate components show excellent fits of > 0.9 for both.

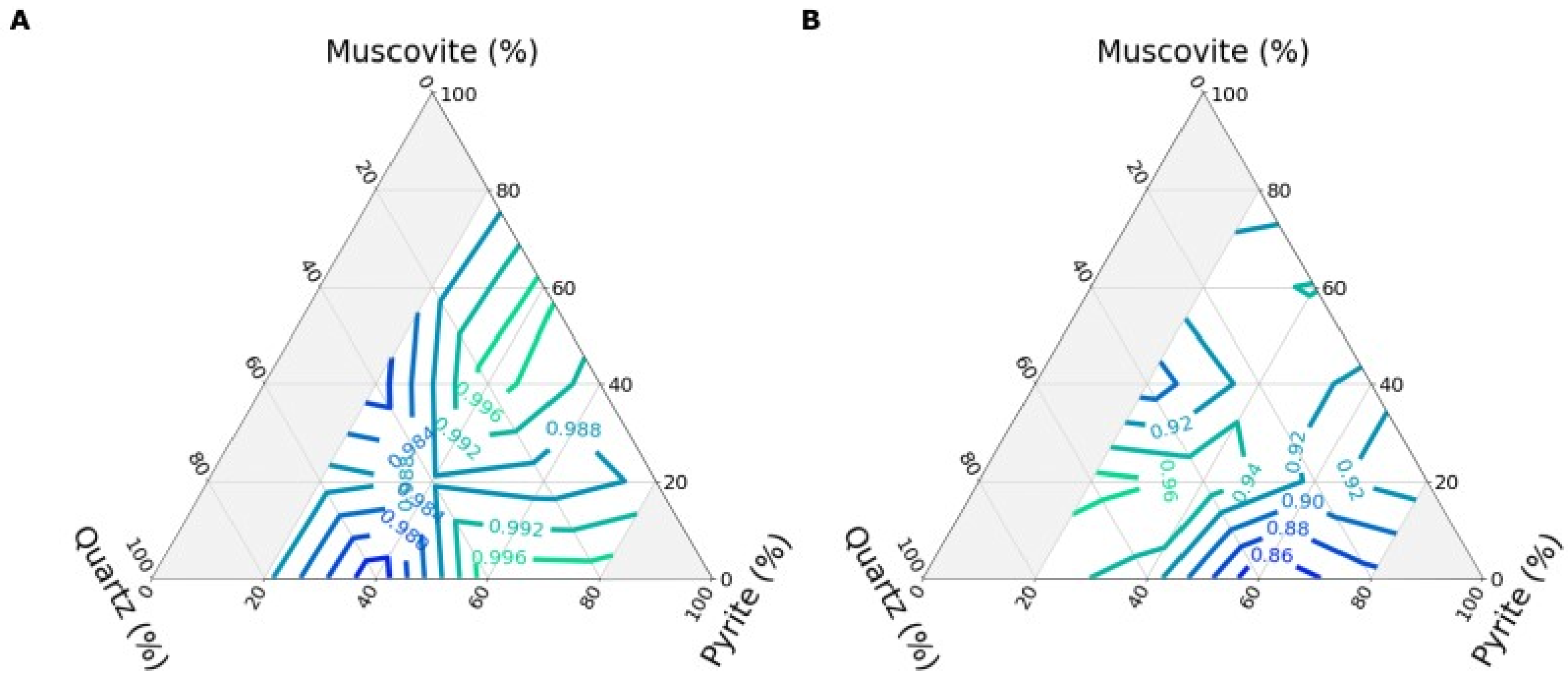

Figure S15: Ternary diagrams of the r² values for the model fit to the differential capacity analysis data. A) r² values fit to the power law model for the pyrite components. B) r² values fit to the linear regression model for the silicate components.

Pre-Cycling Coulombic Efficiency

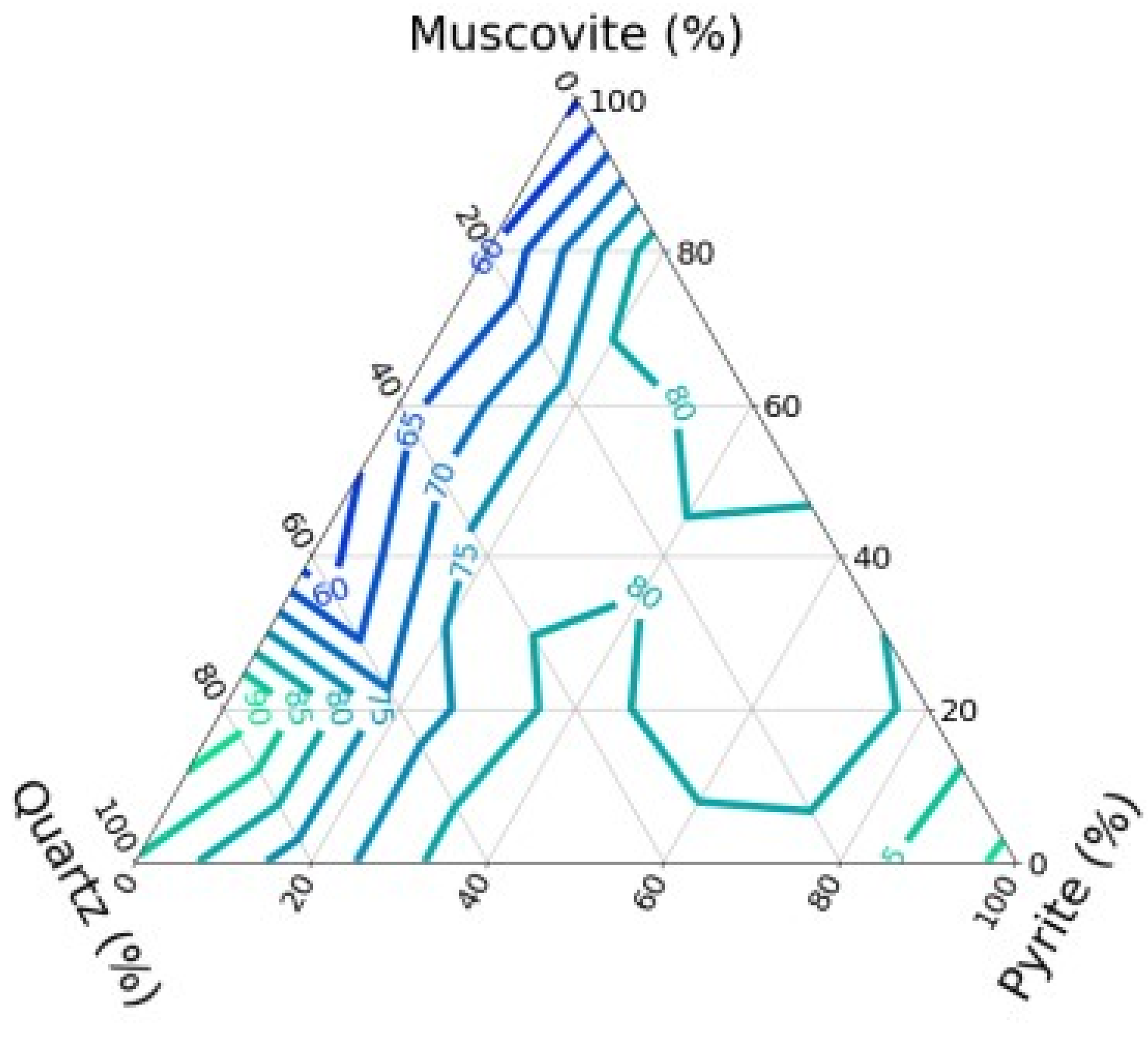

Figure S16: Ternary diagram of the initial Coulombic efficiency for each ternary mixture.

Pre-Cycling R_{ct} Values

Pre-cycling the EIS spectra for each ternary mixture electrode was fit to the equivalent circuit model shown in Figure S17. The respective R_{ct} values generated from the equivalent circuit model are shown in Figure S18.

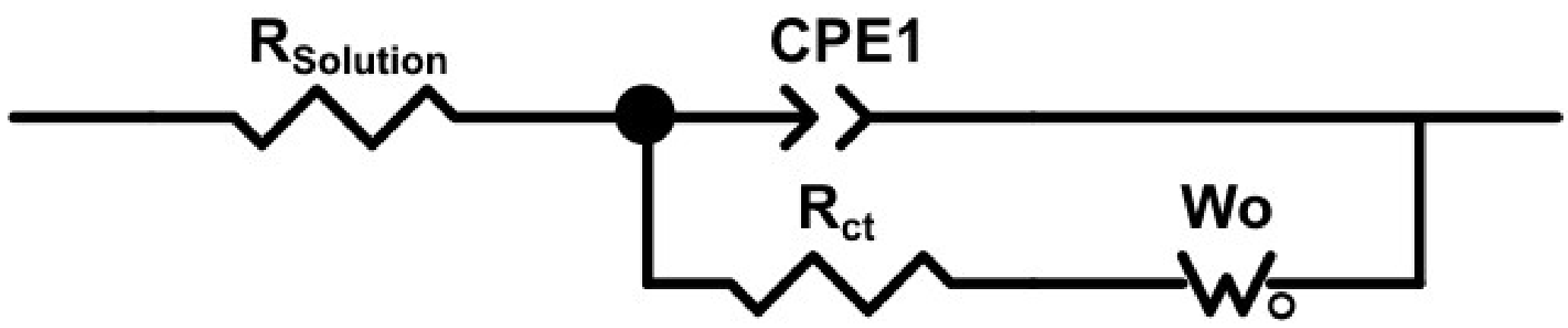

Figure S17: Equivalent circuit model fit to EIS spectra pre-cycling for each ternary mixture anode.

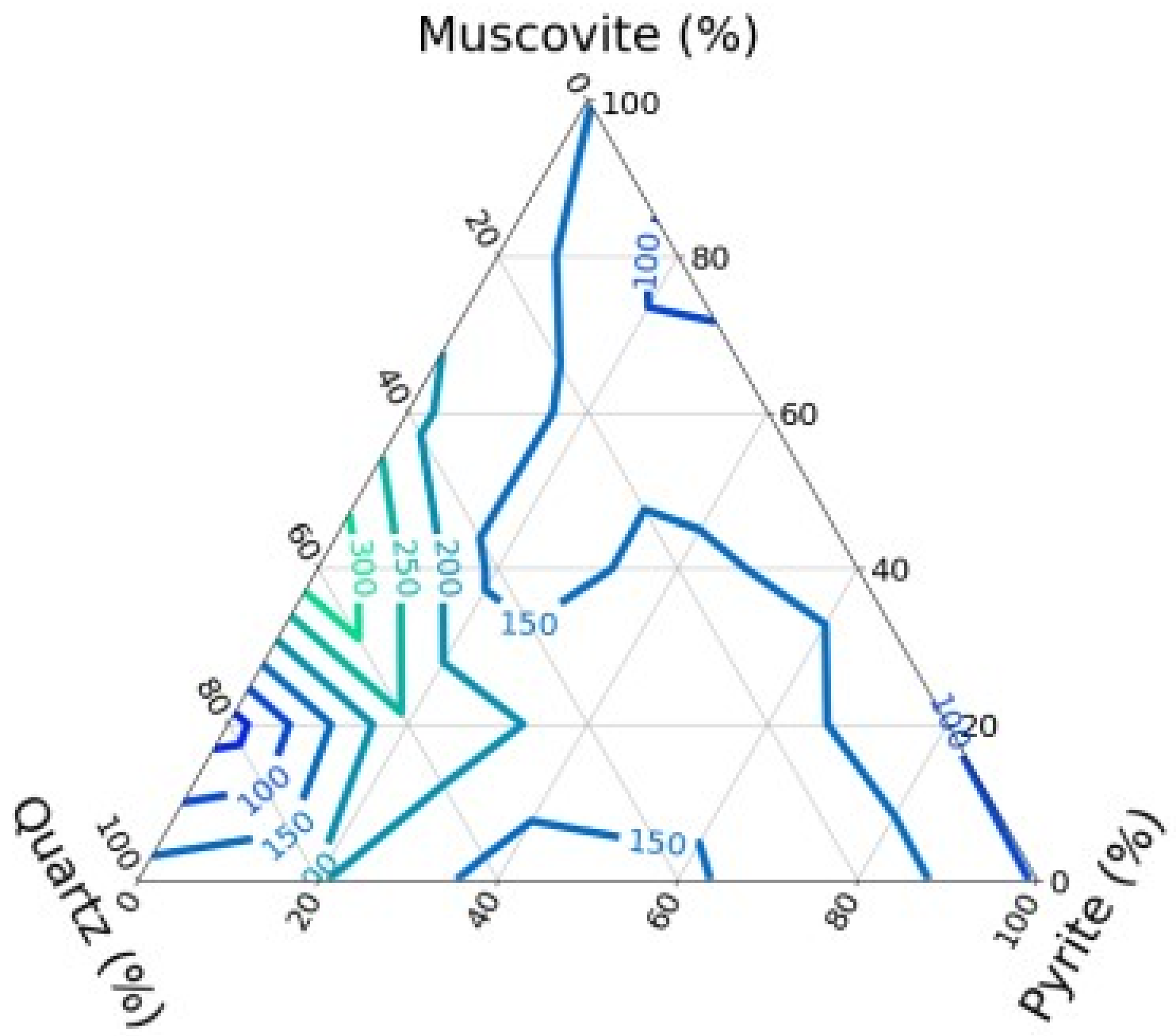

Figure S18: Ternary diagram for the pre-cycling R_{ct} value for the ternary mixtures.

Post-Cycling Modelled Impedance Values

After one hundred cycles at 1C the EIS spectra for each ternary mixture electrode were fit to the equivalent circuit model shown in Figure S19.

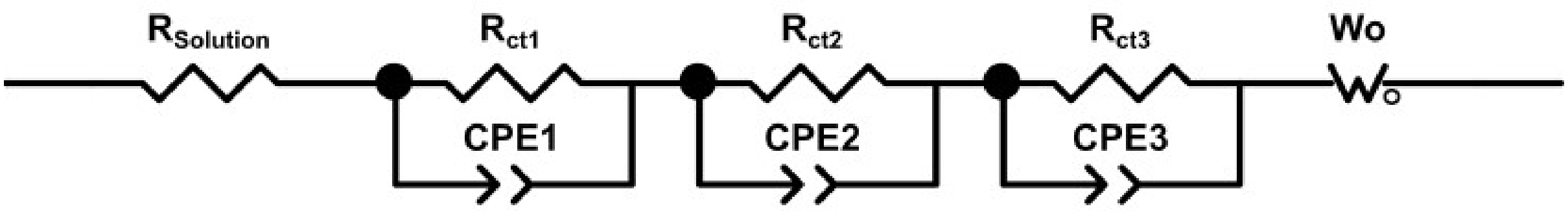

Figure S19: Equivalent circuit model fit to EIS spectra after one hundred cycles at 1C for each ternary mixture anode.

The R_{ct1}, R_{ct2} and R_{ct3} components are shown in the ternary diagrams of Figure S20.

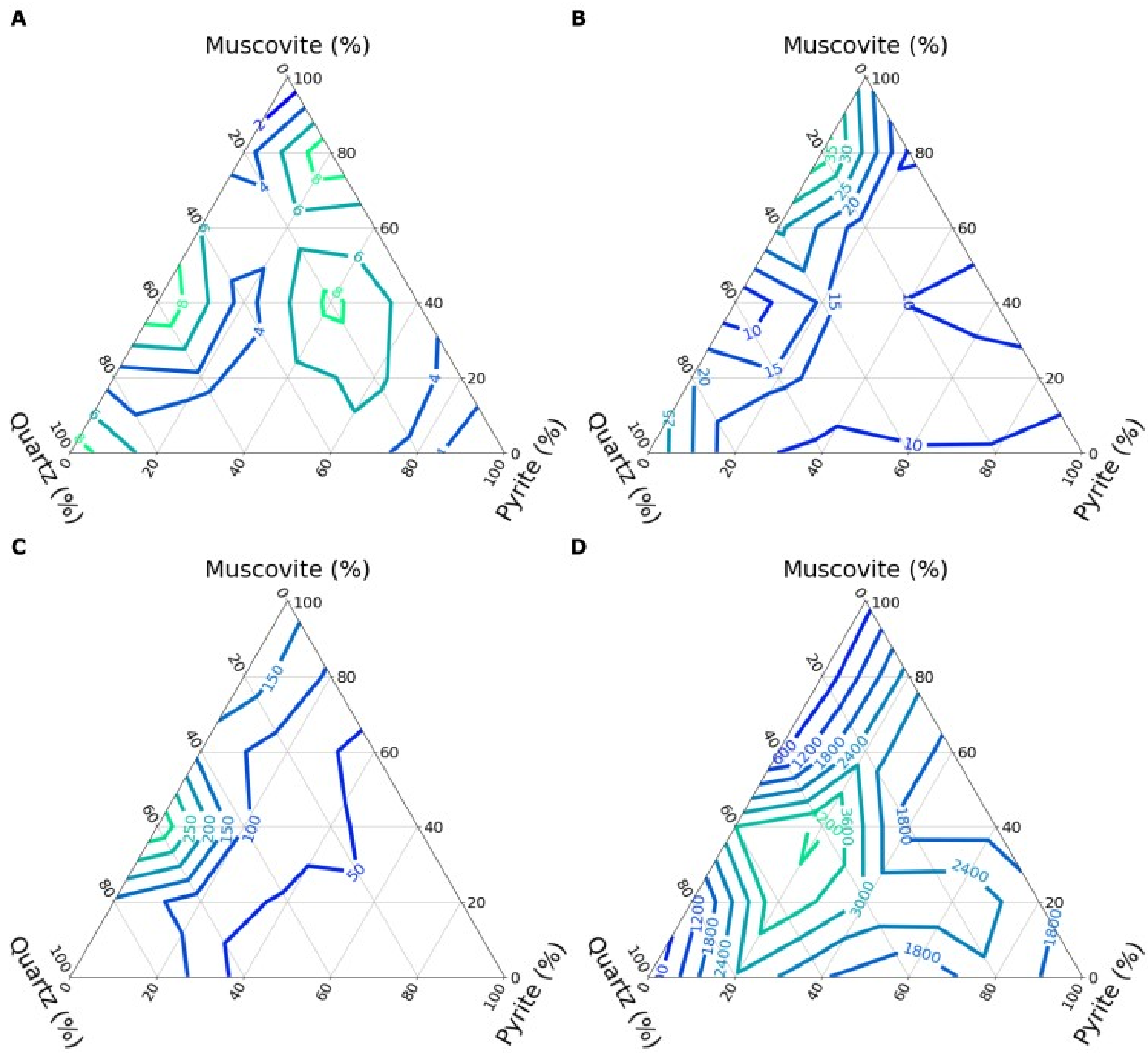

Figure S20: Ternary diagrams for impedance values modelled post-cycling for each ternary mixture. A) $R_{solution}$. B) R_{ct1}, C) R_{ct2} component and D) R_{ct3} component.

D_{Li+} Determination from the Warburg component of EIS spectra

To determine the D_{Li+} value for each ternary mixture anode pre-cycling and post-cycling, the Warburg impedance region was determined for each EIS spectrum as the linear region at low frequencies. Equation S3 was then used to determine the D_{Li+} value as shown below:

$$D = \left(\frac{RT}{\sigma n^2 F^2 A\sqrt{2}C}\right)^2$$

(S3)

A description of the values used is provided below in Table S2:

Table S2: Table of parameters used for determining the DLi+ value from EIS spectra using Equation

Symbol	Name	Value
**R**	Molar gas constant	8.3415 $JK^{-1}mol^{-1}$
**T**	Temperature	293.15 K
**σ**	Warburg factor	Determined from the slope of $Z – \omega^{-0.5}$ plot
**n**	Charge transfer number	1
**F**	Faraday constant	96485 $Cmol^{-1}$
**A**	Electrode area	П*0.14 cm^2
**C**	Li^+ concentration	1 $moldm^{-3}$

S3.

As the Warburg angle was not 45° pre-cycling and post-cycling, as shown in Figures S21B and S22B respectively, the D_{Li+} value was calculated for both the real component of the impedance (Z_{re}) and the imaginary component of the impedance (Z_{im}) and the modulus of this was determined to generate a value for D_{Li+} as shown in Figures S21A and Figures S22A respectively.

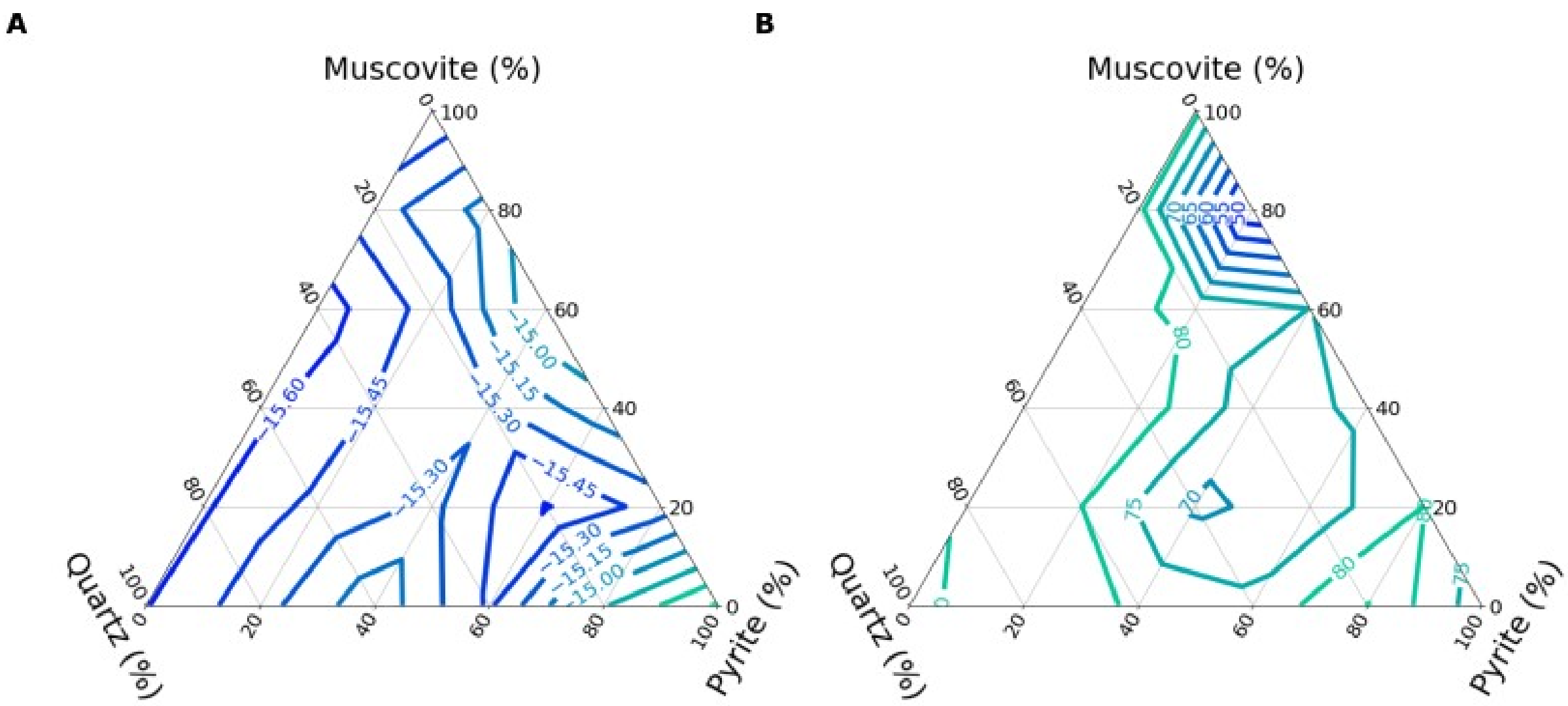

Figure S21: Ternary diagrams for the determination of D_{Li+} values pre-cycling for each ternary mixture. A) Modulus D_{Li+} values. B) Warburg angle.

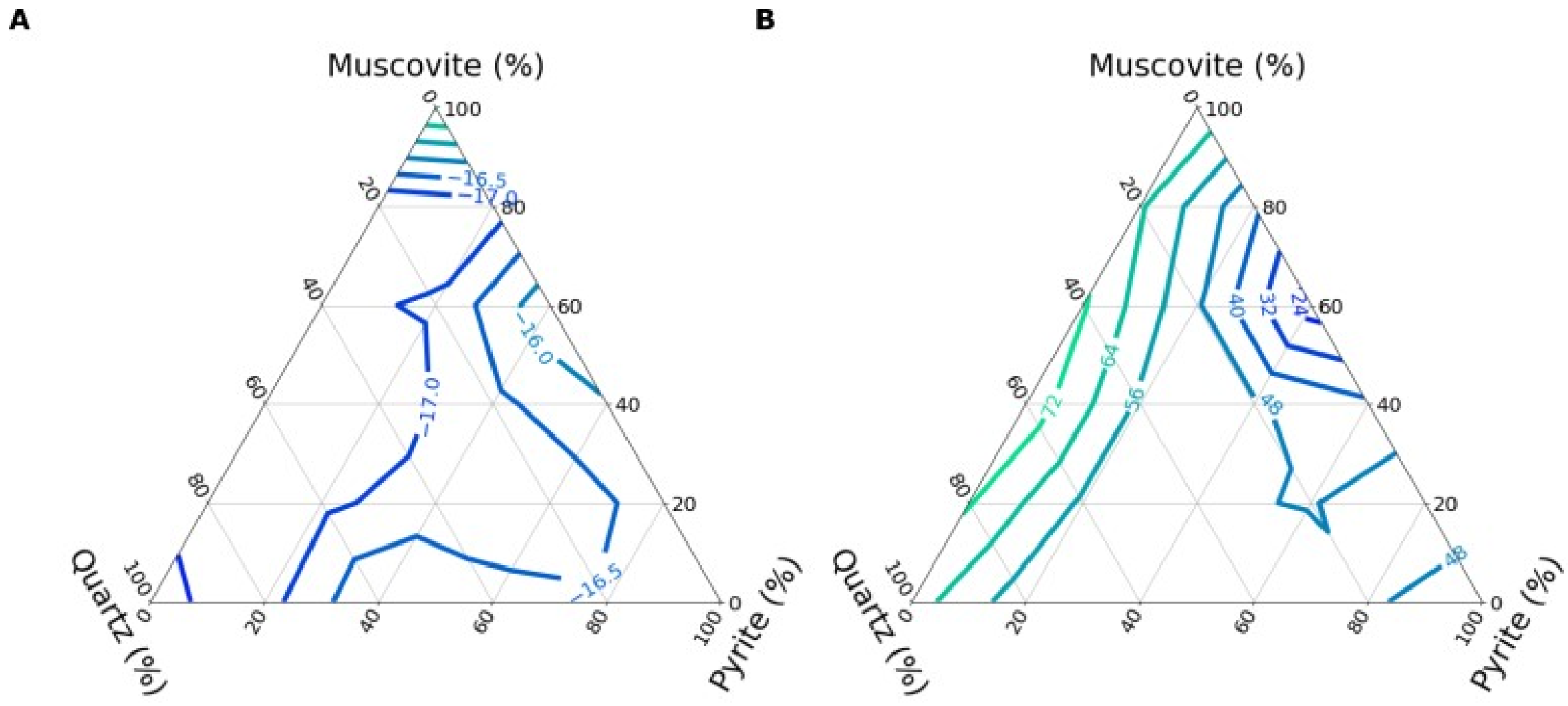

Figure S22: Ternary diagrams for the determination of D_{Li+} values post-cycling for each ternary mixture. A) Modulus D_{Li+} values. B) Warburg angle.

S4: Postmortem Ex-Situ Electrode Analyses

For postmortem analyses, electrodes were extracted from half-cells cycled one hundred times at 1C. Electrodes were removed within an Argon atmosphere within a glovebox and allowed to dry for 1 week before being characterised.

Post-Cycling SEM and EDX Elemental Maps

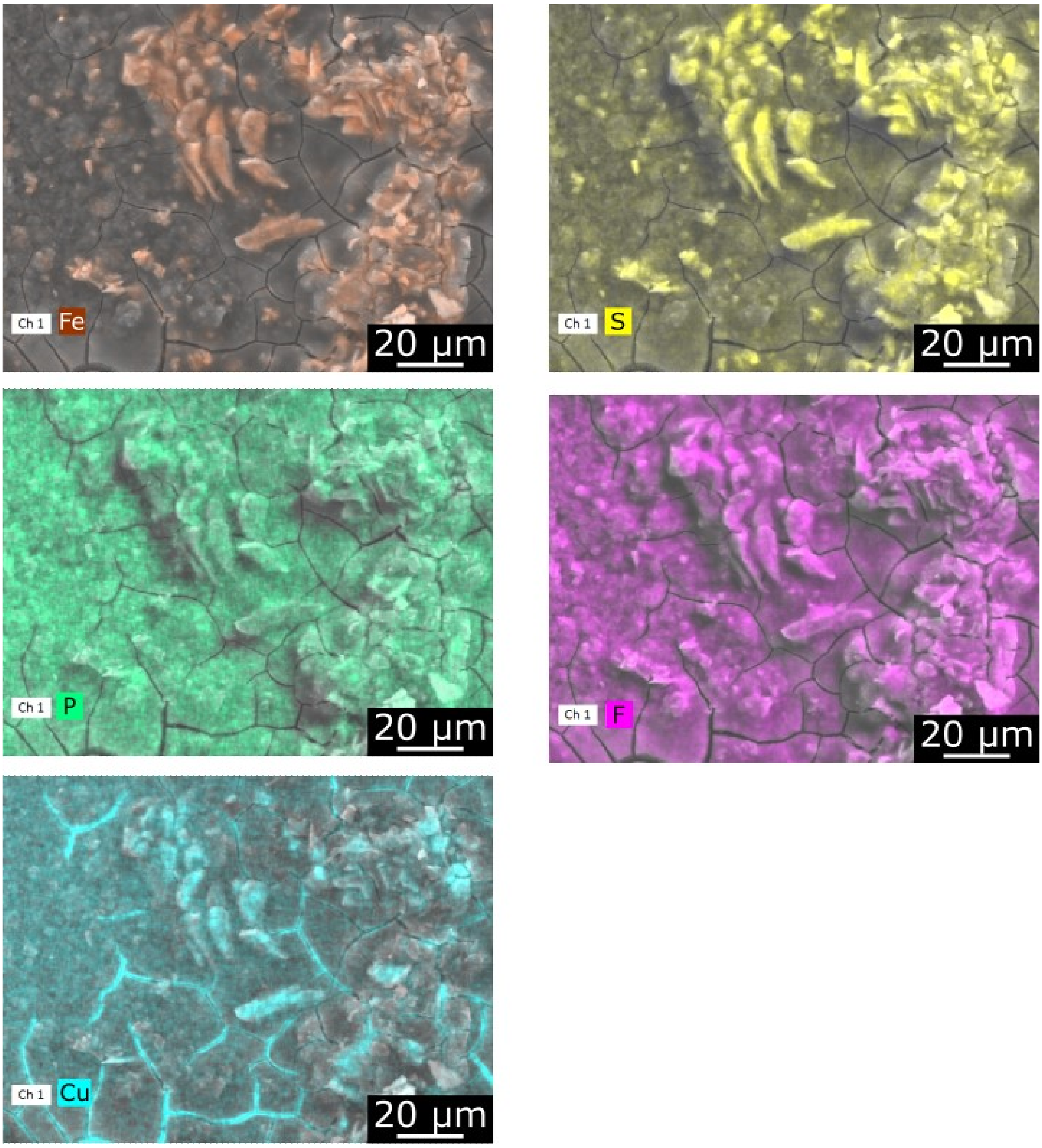

Figure S23: EDX maps for a pyrite electrode post-cycling for the elements: Fe, S, P, F and Cu.

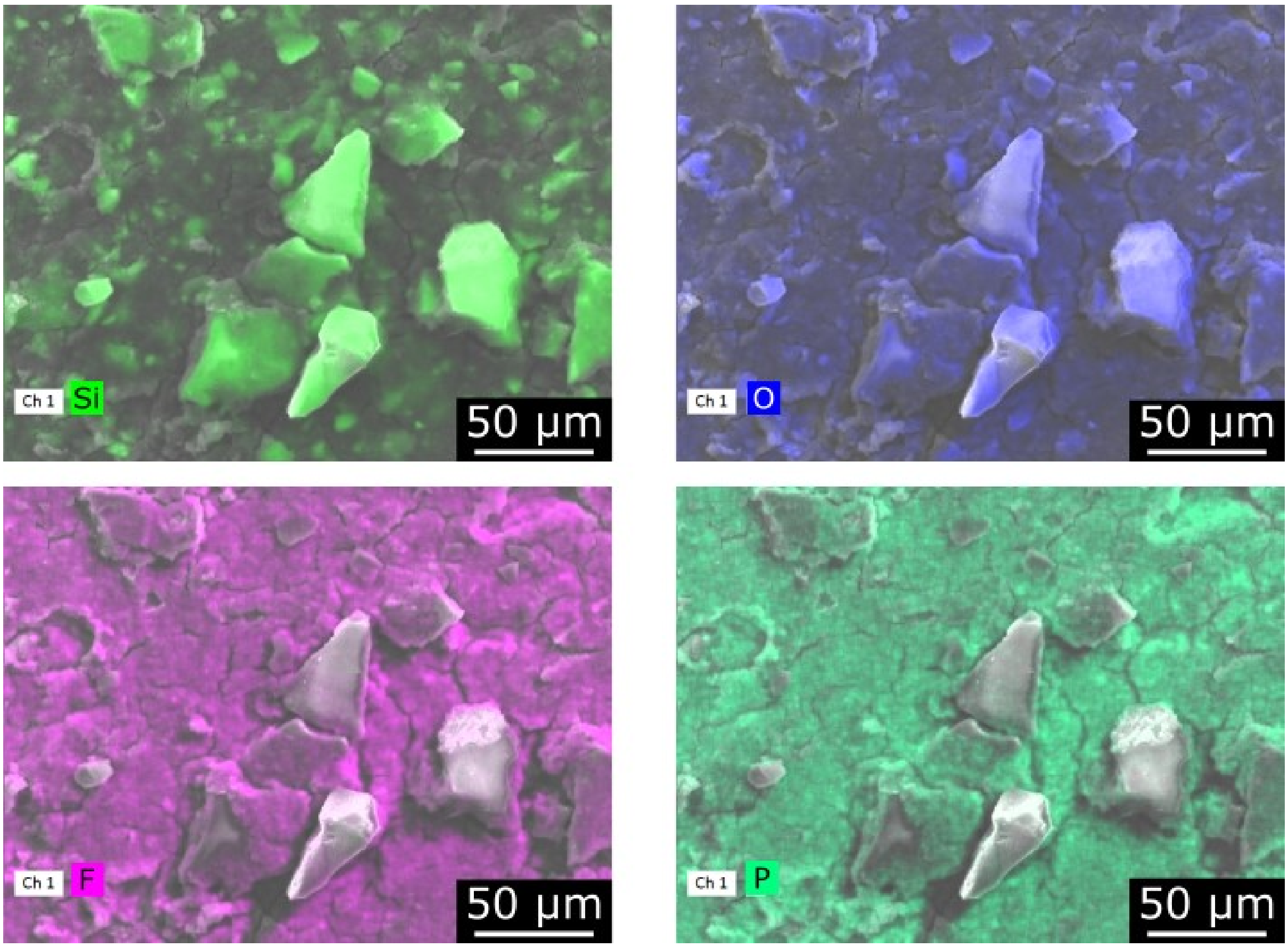

Figure S24: EDX maps for a quartz electrode post-cycling for the elements: Si, O, F and P.

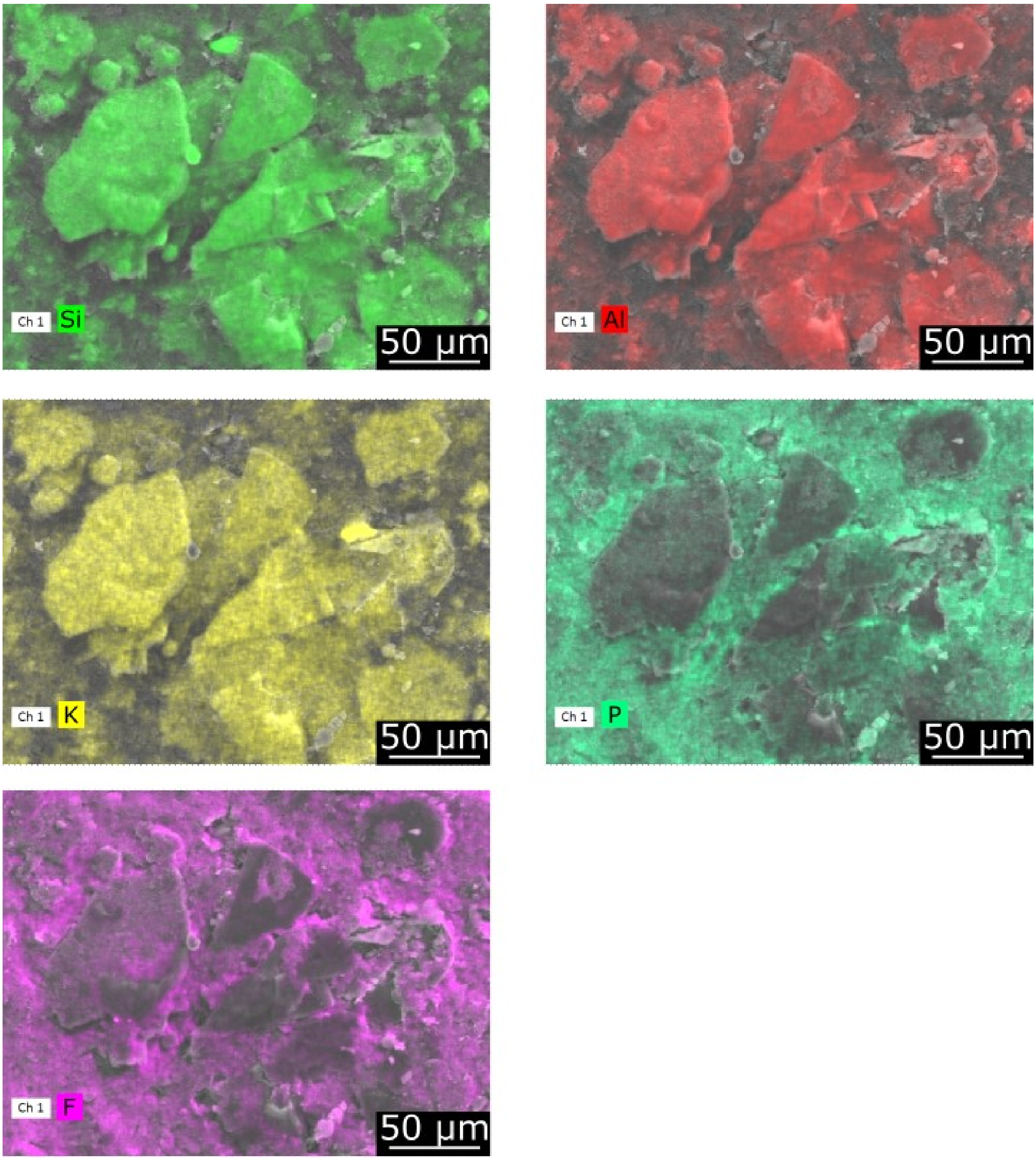

Figure S25: EDX maps for a muscovite electrode post-cycling for the elements: Si, Al, K, P and F.

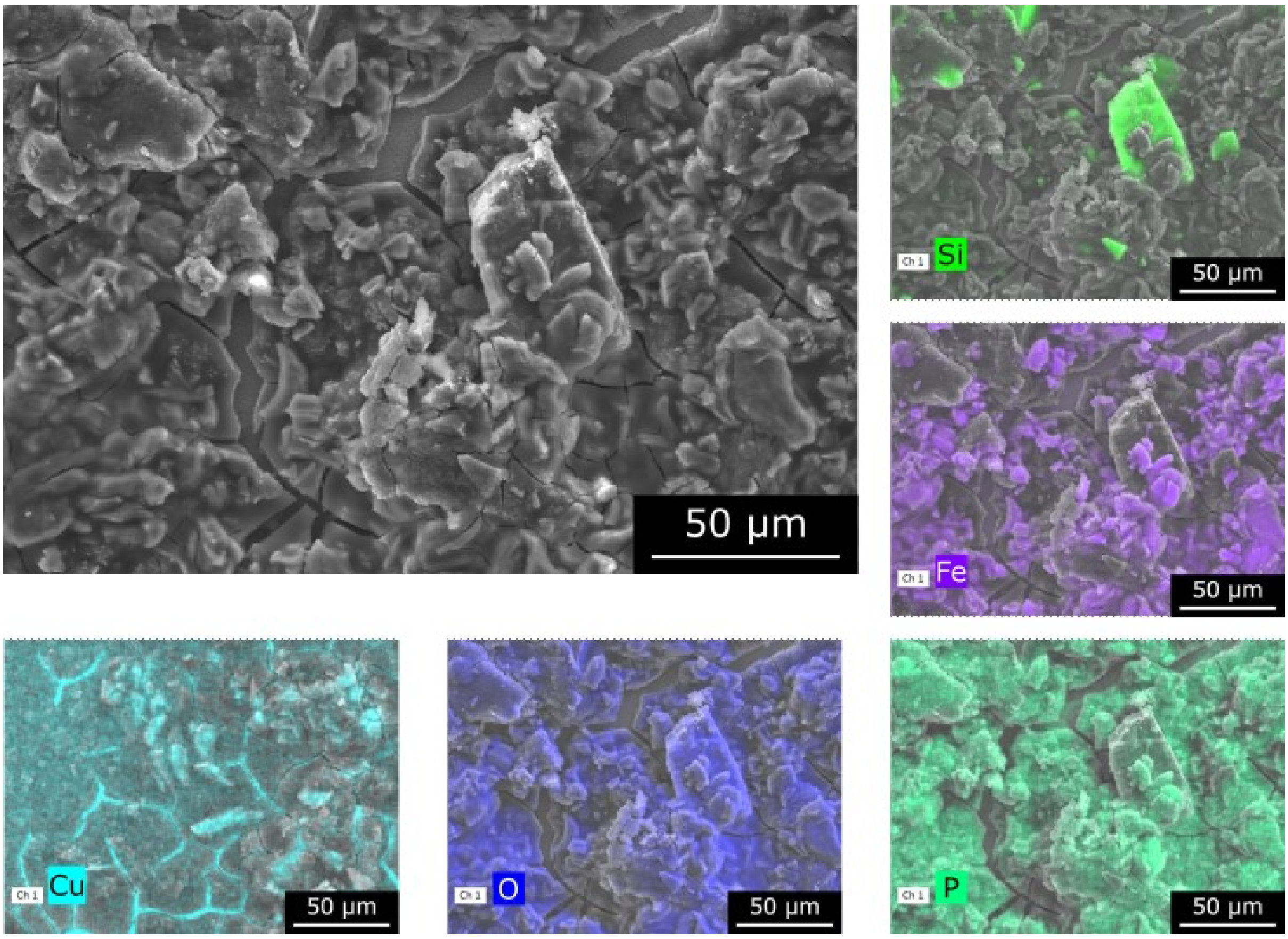

Figure S26: SEM image and EDX map for a 60% pyrite and 40% quartz electrode post-cycling. Elemental maps are shown for Si, Fe, Cu, O and P.

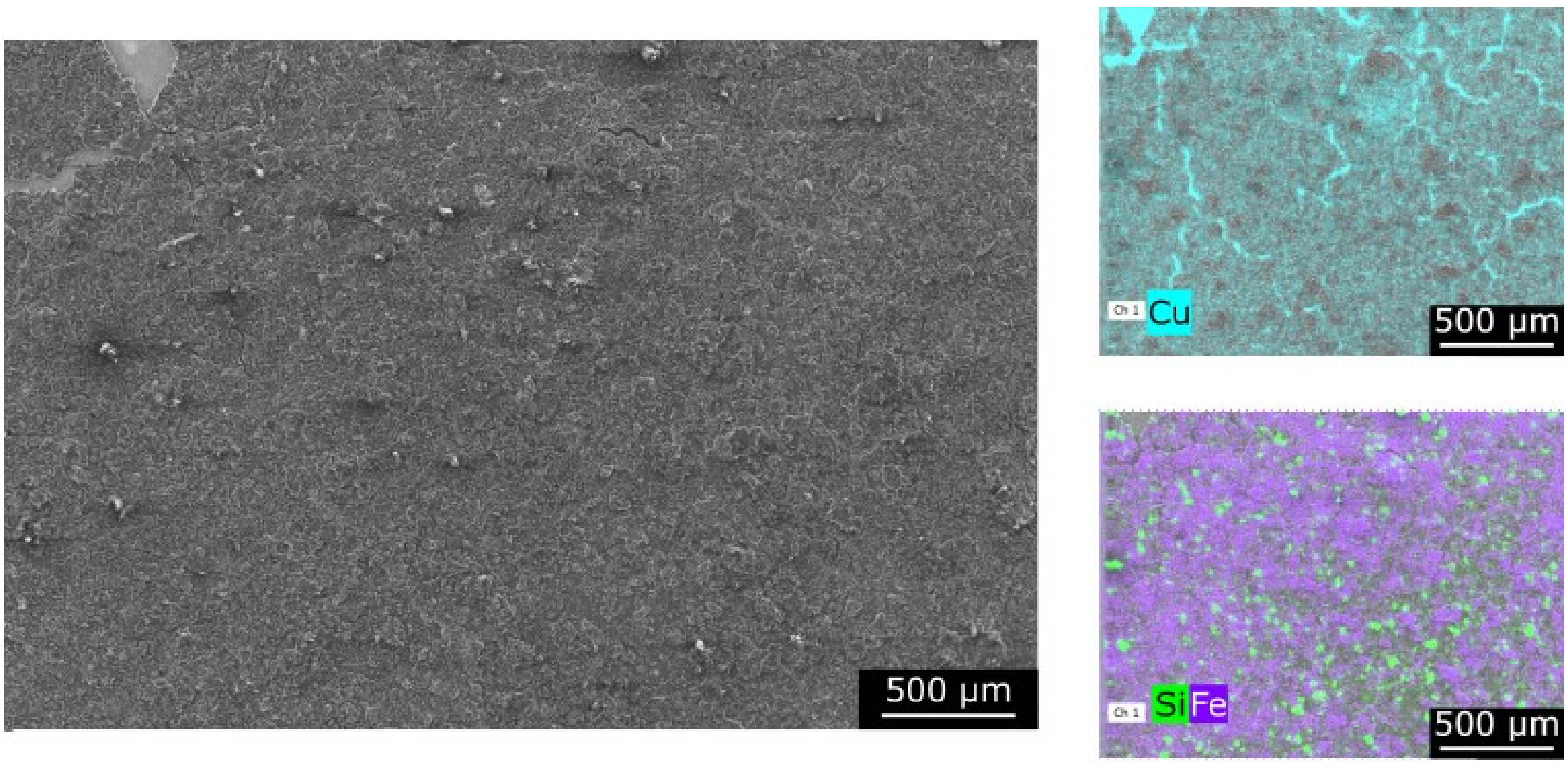

Figure S27: SEM image and EDX map for a 60% pyrite and 40% quartz electrode post-cycling. Elemental maps are shown for Cu, Si and Fe.

XRD Patterns

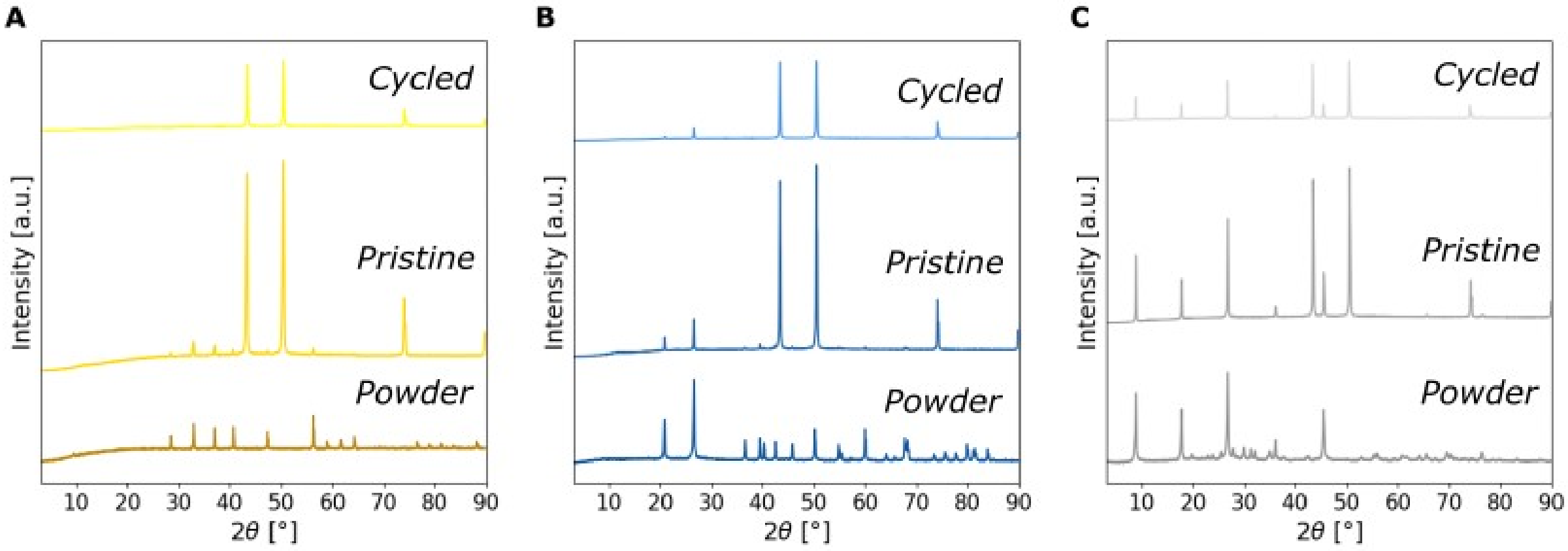

Figure S28: XRD patterns of the 2θ range 5º – 90º of the pure phase minerals as a powder, pristine electrode and cycled electrode. A) Pyrite. B) Quartz. C) Muscovite.

TEM and STEM Imagery

For TEM and STEM imagery attained, extracted electrodes had their slurry material removed from the copper foil they were cast on and placed in a vial of Iso Propyl Alcohol (IPA), then sonicated for 3 minutes. Particles were then drop cast from this solution onto a copper grid covered with a holey carbon film.

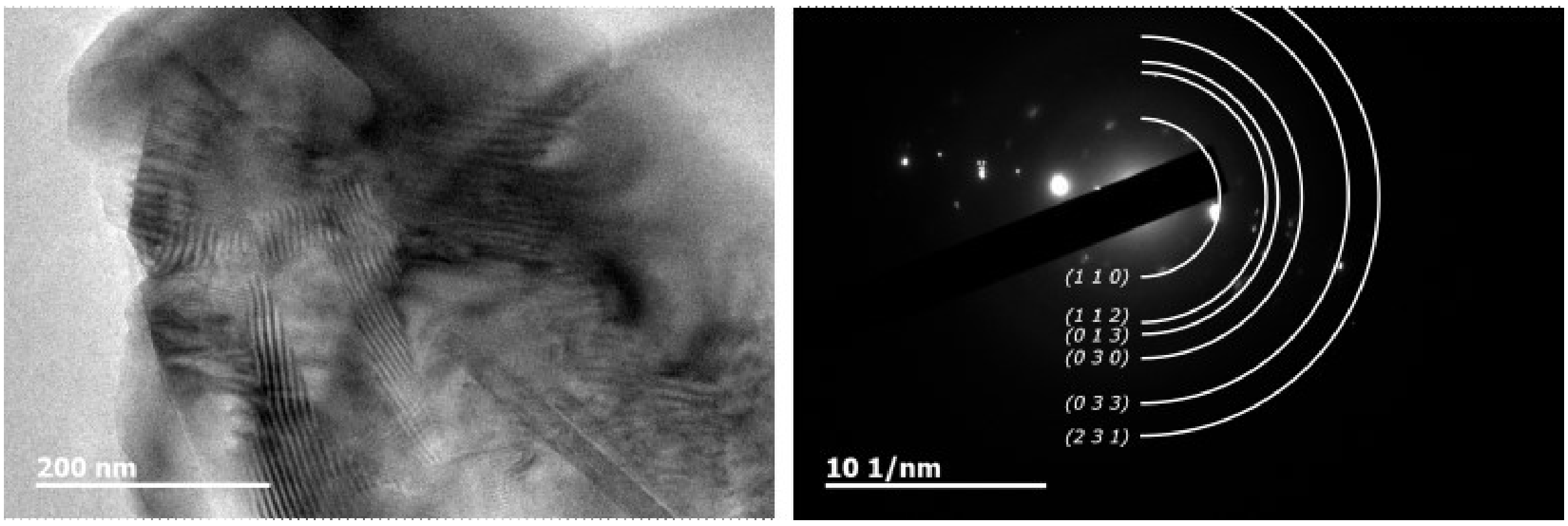

Figure S29: TEM image of a quartz particle pre-cycling with its corresponding SAED image with rings indexed for quartz.

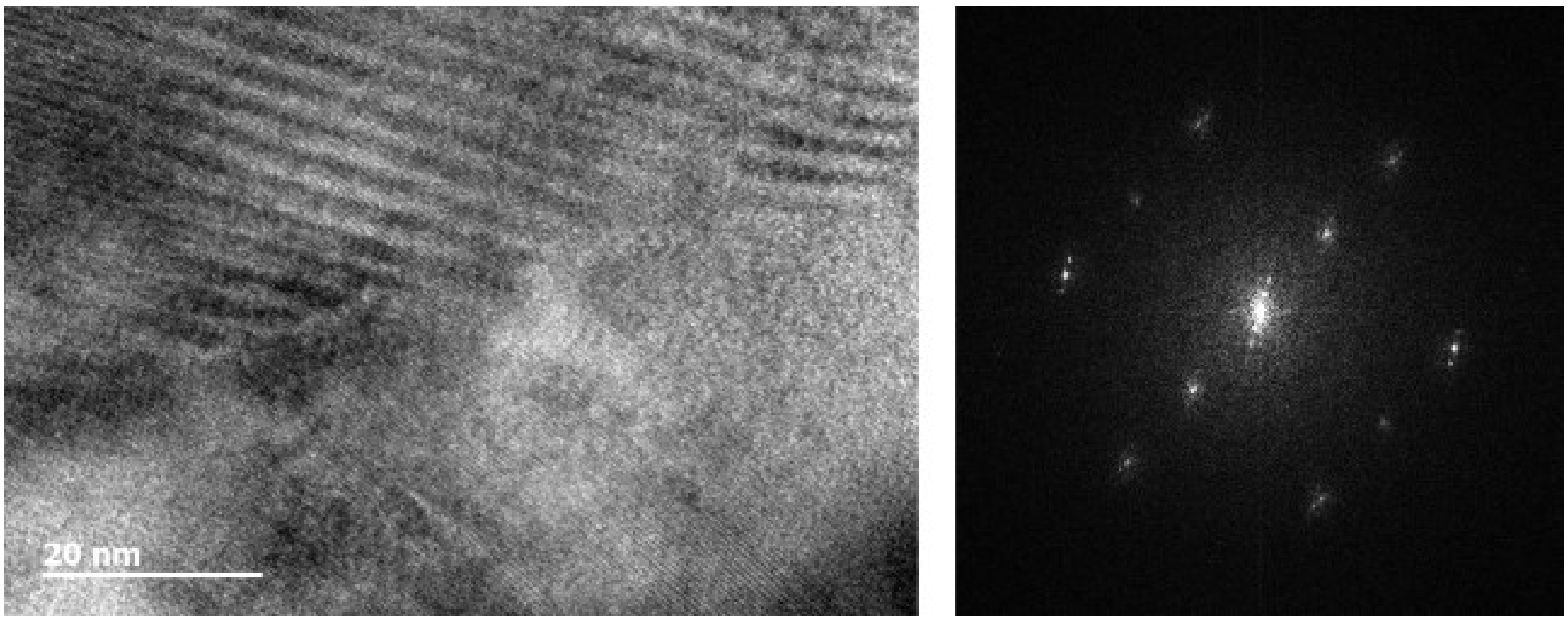

Figure S30: TEM and corresponding FFT image of a quartz particle pre-cycling.

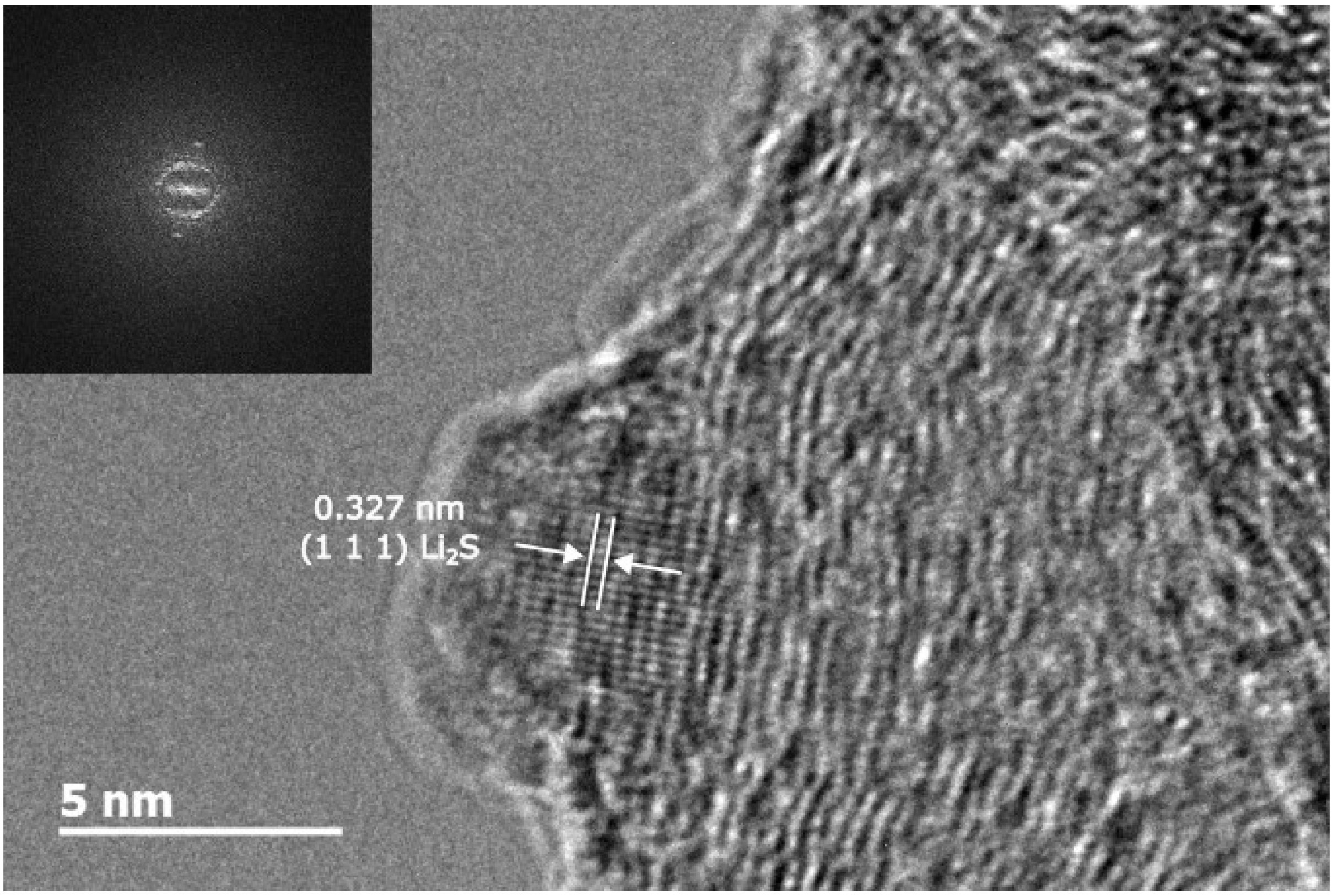

Figure S31: TEM image of an indexed particle of Li_2S nano nodule on a particle of super p carbon black post-cycling. Insert is the corresponding FFT image.

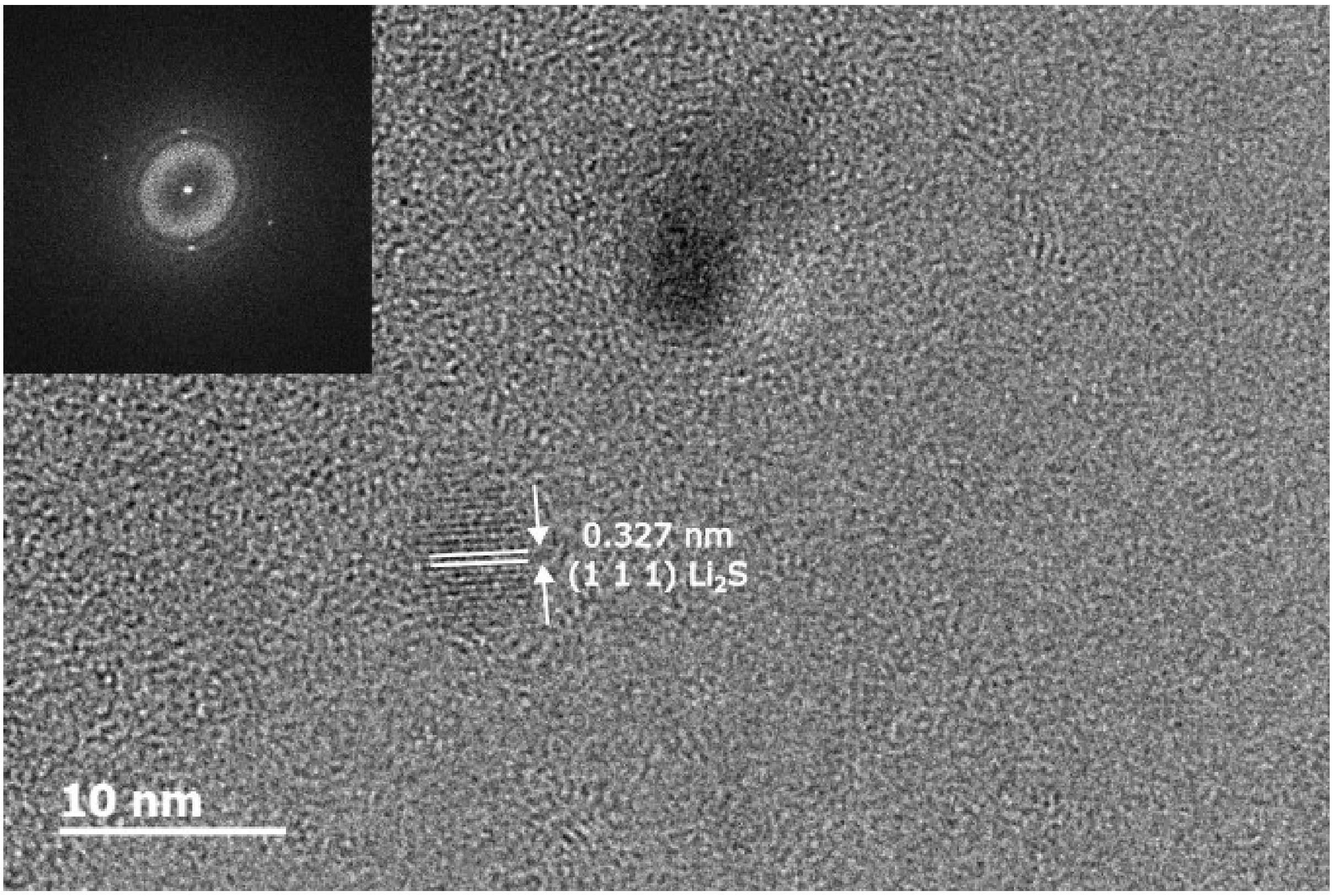

Figure S32: TEM image of an indexed particle of Li_2S nano nodule on a non-crystalline pyrite particle post-cycling. Insert is the corresponding FFT image.

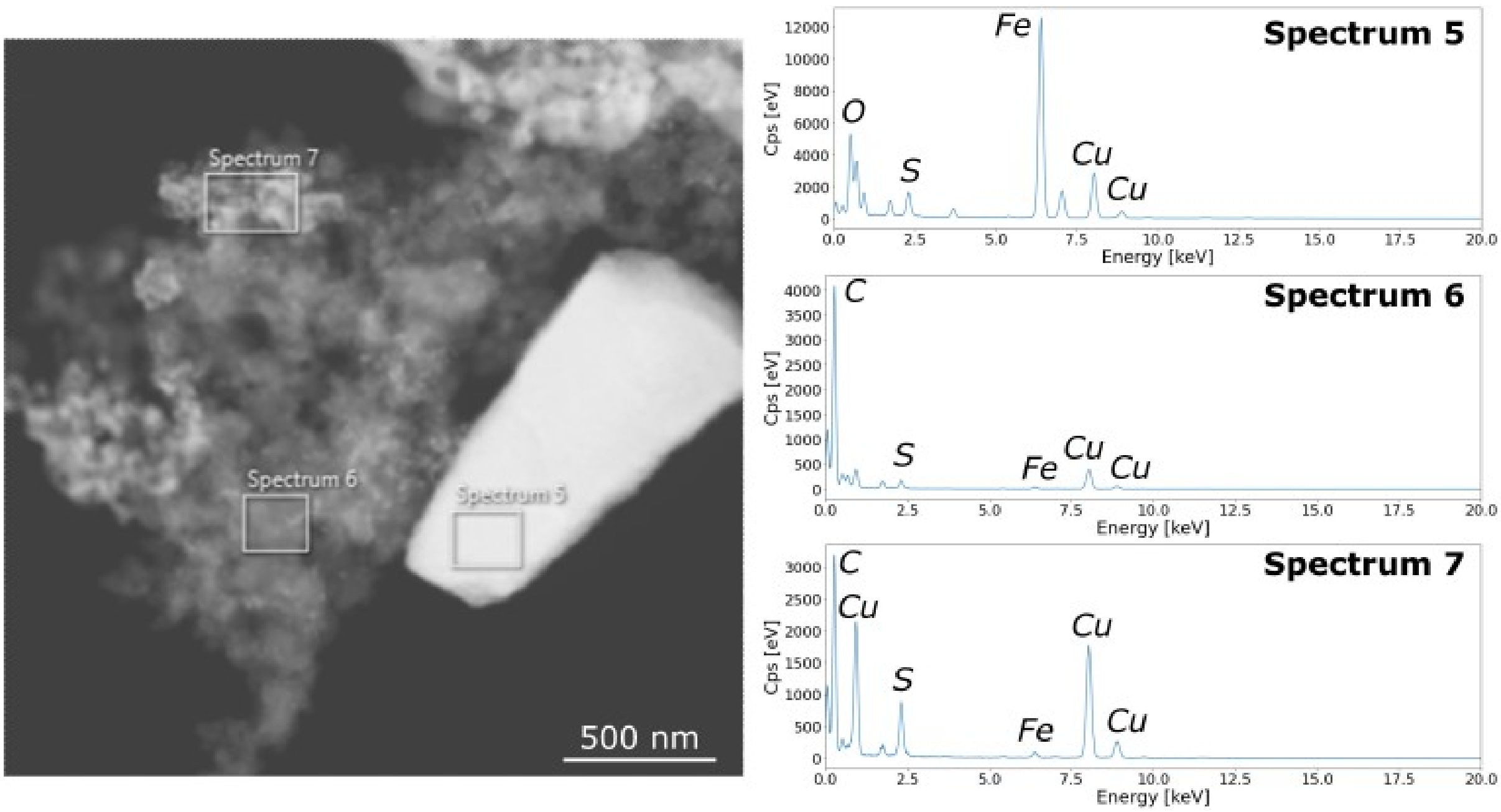

Figure S33: STEM image of a pyrite particle and surrounding matter post-cycling. Spectra from STEM of selected areas are shown.

XRD Rietveld Refinement

Rietveld refinement was undertaken on XRD patterns ascertained for the pyrite, quartz and muscovite powder, pristine electrode and electrode after 100 cycles at 0.1C. XRD patterns were fitted using the pyrite, quartz, muscovite 2M1 and copper structure files inbuilt within the Profex software used to undertake the refinement. The fitted parameters for each refinement are provided in Table S3*.*

Table S3: Refined parameters from XRD for powders, pristine electrodes and cycled electrodes for pyrite, quartz and muscovite.

	Pyrite powder	Pyrite pristine electrode	Pyrite cycled electrode	Quartz powder	Quartz pristine electrode	Quartz cycled electrode	Muscovite powder	Muscovite pristine electrode	Muscovite cycled electrode
**a [nm]**	0.5412	0.54211	N/A	0.49102	0.49191	0.49106	0.5181	0.5159	0.5236
**b [nm]**	N/A	N/A	N/A	N/A	N/A	N/A	0.9012	0.8903	0.8904
**c [nm]**	N/A	N/A	N/A	0.54021	0.5415	0.5427	2.00104	2.00393	2.0013
**β [º]**	N/A	N/A	N/A	N/A	N/A	N/A	95.716	96.6469	95.56
**V [Å³]**	159	159	N/A	113	113	113	930	914	929
**Χ²**	1.26	6.94	7.7	4.94	15.15	11.76	4.4	13.36	17.44

Mine Tailings Mineralogy

Machine Liberation Analysis (MLA) was undertaken on over 200, 000 particles for both mine tailings samples using SEM EDX. Table S3 and Table S4 provide the mineralogy for the HP and LP tailings, respectively.

**Mineral**	**Wt%**
**Pyrite**	81.40%
**Pyrite Mineral Mixes**	17.44%
**Quartz**	0.68%
**Trace Mineral Phases**	0.48%

Table S4: Modal mineralogy by weight percentage for the HP tailings.

Table S5: Modal mineralogy by weight percentage for the LP tailings.

Mineral	Wt%%
**Orthoclase Feldspar**	20.76%
**Quartz**	17.94%
**Pyrite**	16.03%
**Micas**	11.65%
**Metal Oxides**	11.08%
**Other Feldspar**	8.76%
**Salts (Carbonates and Phosphates)**	4.62%
**Amphiboles**	4.39%
**Garnets**	3.47%
**Trace Mineral Phases**	1.30%
